# Extrahypothalamic GABAergic nociceptin–expressing neurons regulate AgRP neuron activity to control feeding behavior

**DOI:** 10.1172/JCI130340

**Published:** 2019-11-18

**Authors:** Mark A. Smith, Agharul I. Choudhury, Justyna A. Glegola, Paulius Viskaitis, Elaine E. Irvine, Pedro Caldas Custodio de Campos Silva, Sanjay Khadayate, Hanns Ulrich Zeilhofer, Dominic J. Withers

**Affiliations:** 1 Institute of Clinical Sciences, Faculty of Medicine, Imperial College, London, United Kingdom.; 2 MRC London Institute of Medical Sciences, London, United Kingdom.; 3 Institute of Pharmacology and Toxicology, University of Zurich, Zurich, Switzerland.; 4 Institute of Pharmaceutical Sciences, Swiss Federal Institute of Technology (ETH) Zurich, Zurich, Switzerland.

**Keywords:** Metabolism, Neuroscience, Melanocortin, Neuroendocrine regulation, Obesity

## Abstract

Arcuate nucleus agouti–related peptide (AgRP) neurons play a central role in feeding and are under complex regulation by both homeostatic hormonal and nutrient signals and hypothalamic neuronal pathways. Feeding may also be influenced by environmental cues, sensory inputs, and other behaviors, implying the involvement of higher brain regions. However, whether such pathways modulate feeding through direct synaptic control of AgRP neuron activity is unknown. Here, we show that nociceptin-expressing neurons in the anterior bed nuclei of the stria terminalis (aBNST) make direct GABAergic inputs onto AgRP neurons. We found that activation of these neurons inhibited AgRP neurons and feeding. The activity of these neurons increased upon food availability, and their ablation resulted in obesity. Furthermore, these neurons received afferent inputs from a range of upstream brain regions as well as hypothalamic nuclei. Therefore, aBNST GABAergic nociceptin neurons may act as a gateway to feeding behavior by connecting AgRP neurons to both homeostatic and nonhomeostatic neuronal inputs.

## Graphical abstract

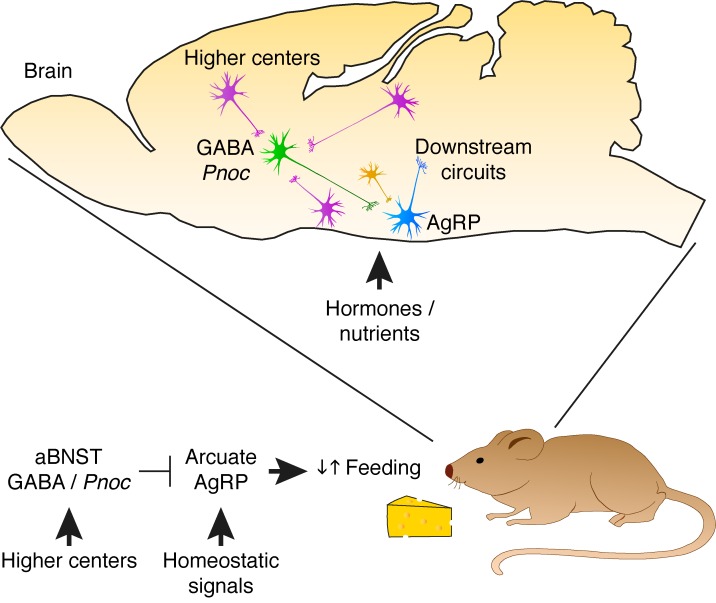

## Introduction

Feeding requires the integration of multiple neuronal networks to elicit appropriate behavioral and metabolic responses (1–3). These networks are sensitive to a range of modulatory inputs including nutrients and hormones, the reward value of food, environmental cues, and the internal emotional state (1–3). Although multiple anatomically defined regions and chemically defined neurons control feeding, hypothalamic arcuate nucleus agouti–related protein–expressing (AgRP-expressing) and neuropeptide Y–expressing (NPY-expressing) neurons (AgRP/NPY neurons) play a central role in feeding and related behaviors (4–6). AgRP/NPY neurons are themselves under complex regulation by peripheral hormonal and nutrient signals and by neuronal inputs including those from the paraventricular hypothalamus (PVN) and dorsomedial nucleus of the hypothalamus (DMH) (1–3, 7, 8). AgRP/NPY neuronal excitability increases during nutrient deficiency and falls upon food presentation and consumption and involves specific postingestive signals and intrahypothalamic circuits (7, 9–13). A range of other inputs such as sensory cues, reward signals, stress, learning, and memory have an impact on AgRP/NPY neuron activity, implying that they may also be regulated by neuronal circuits from outside the hypothalamus (7, 10, 13, 14). However, to date, no extrahypothalamic afferent pathways have been identified that directly synaptically regulate AgRP/NPY neuron activity and hence feeding. Therefore, we sought to explore potential circuits that might directly regulate AgRP/NPY neurons, focusing on the bed nuclei of the stria terminalis (BNST), as neuronal tracing studies have demonstrated anatomical connections between the BNST and the arcuate nucleus, and the BNST is involved in appetitive feeding behaviors (15–21).

Here, we show that anterior BNST (aBNST) neurons coexpressing GABA and the opioid-like peptide nociceptin (a) inhibited arcuate AgRP/NPY neurons via GABAergic transmission to suppress food intake independent of changes in anxiety; (b) showed increases in endogenous activity at the initiation of feeding, which was temporally consistent with the reduction in AgRP/NPY activity; (c) regulated body weight; and (d) received afferent inputs from a range of upstream brain regions. These studies thus identify aBNST GABAergic nociceptin neurons as a previously unrecognized extrahypothalamic neuron population that regulates food intake and body weight via AgRP/NPY neurons by acting as a potential feeding gateway to link both homeostatic and nonhomeostatic inputs.

## Results

### GABAergic neurons in the aBNST directly inhibit arcuate NPY neurons.

**Figure 1:**
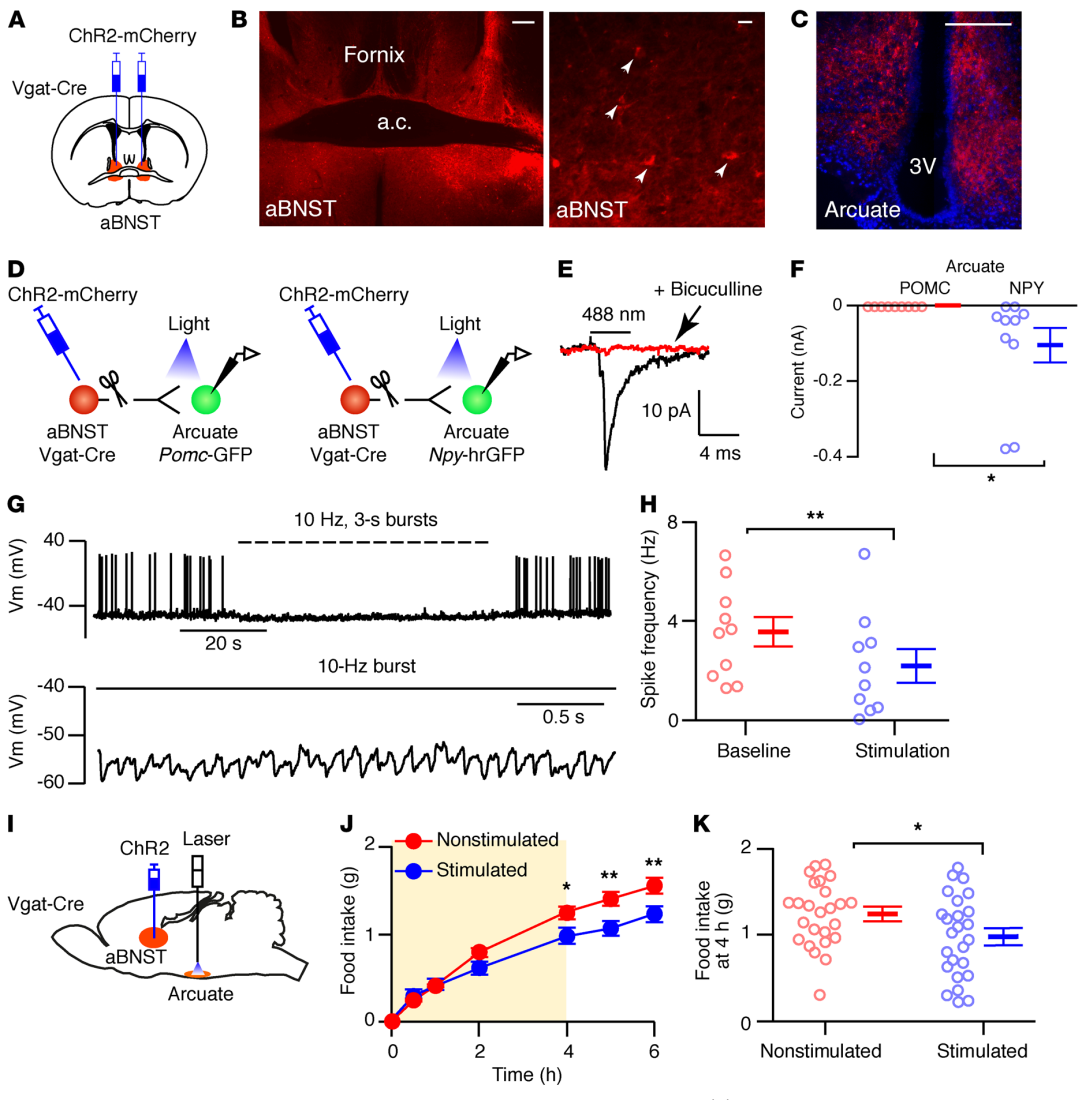
Photostimulation of aBNST GABAergic axons in the arcuate nucleus suppresses feeding. (**A**) Diagram illustrating injections of the aBNST (red) with ChR2-mCherry. (**B**) Mosaic images (*n* = 35) of aBNST (enlarged inset on the right) and arcuate nucleus (**C**) from Vgat-Cre mice with ChR2-mCherry AAV injected into the aBNST. Scale bars: 200 μm (low-magnification images) and 20 μm (high-magnification images). (**D**) Diagram of Vgat-Cre mice crossed with *Pomc*-GFP or *Npy*-hrGFP mice with ChR2-mCherry in the aBNST. (**E**) Photostimulated synaptic currents from an arcuate NPY neuron in the absence (black) and presence (red) of 20 μM bicuculline (*n* = 10). (**F**) Synaptic currents in arcuate POMC (*n* = 9 neurons from 3 mice) and NPY (*n* = 10 neurons from 3 mice) neurons. Data represent the mean ± SEM. *P < 0.05 [unpaired *t* test, *t* (17) = 2.15, *P* = 0.046]. (**G**) Voltage traces (expanded below) from a NPY neuron during photostimulation of aBNST axons (*n* = 10). Vm, membrane potential. (**H**) Action potential frequency in NPY neurons (*n* = 10 neurons from 3 mice) before (baseline) and during photostimulation of aBNST axons. Data represent the mean ± SEM. **P < 0.01, by paired *t* test, *t* (9) = 3.33, *P* = 0.009. (**I**) Diagram illustrating injection of the aBNST with ChR2-mCherry and optical fiber implantation into the arcuate nucleus. (**J**) Cumulative food intake in fasted Vgat-Cre mice during (shaded area) and after photostimulation of ChR2-mCherry–expressing axons from the aBNST. Food intake was measured in mice without (red) and with (blue) photostimulation. Data represent the mean ± SEM. *n* = 25 mice. Two-way repeated-measures ANOVA [interaction: *f* (6,288) = 9.58, *P* < 0.0001; stimulation: *f* (1,48) = 3.68, *P* = 0.06]. *P < 0.05 and **P < 0.01, by Sidak’s post hoc test. (**K**) Food intake at 4 hours from nonstimulated and stimulated mice. Data represent the mean ± SEM. *n* = 25 mice. *P < 0.05, by paired *t* test, *t* (24) = 2.46, *P* = 0.021. 3V, third ventricle; a.c., anterior commissure.

Anatomical tracing studies have identified axons that project from the anteromedial BNST (amBNST) toward the arcuate nucleus (16, 20, 21). To investigate the function of these projections, we performed virus-based tracing experiments. As BNST neurons are predominantly GABAergic (22), we injected adeno-associated virus (AAV) particles containing a Cre-dependent channelrhodopsin-2-mCherry (ChR2-mCherry) construct into the aBNST of Vgat-Cre mice expressing Cre recombinase driven by the vesicular GABA transporter (*Slc32a1* or Vgat) promoter to selectively transduct GABAergic neurons (Figure 1A). We detected expression of mCherry in somas and fibers surrounding the anterior commissure, thus capturing the various subnuclei of the aBNST (Figure 1B). Furthermore, we also observed mCherry-containing projection fibers in a number of brain regions, with particularly dense expression in the arcuate nucleus (Figure 1C and data not shown). We did not find mCherry-expressing somas in the arcuate nucleus, suggesting that the AAV1 serotype virus particles injected into the aBNST did not retrogradely label neurons in the arcuate nucleus.

To determine whether these aBNST axons made direct synaptic connections with specific arcuate nucleus neurons that regulate feeding, we injected ChR2-mCherry AAVs into the aBNST of Vgat-Cre mice that were crossed with either *Pomc*-topaz GFP–transgenic (*Pomc*-GFP) or *Npy*-*Renilla* GFP–transgenic (*Npy*-hrGFP) mice to identify arcuate pro-opiomelanocortin (POMC) or NPY neurons, respectively. Arcuate nucleus slices that did not contain the BNST but retained ChR2-mCherry–expressing axons from the aBNST were prepared for ex vivo ChR2-assisted circuit mapping (Figure 1D). Synaptic currents were evoked by 4-ms light pulses, and a mean of 80 consecutive trials were used to distinguish photostimulated events from spontaneously occurring synaptic input. We observed an inward current in NPY but not POMC neurons (Figure 1, E and F), which, when tested, was completely blocked by a GABA_A_ receptor antagonist (*n* = 3 neurons from 2 mice, Figure 1E). Moreover, using a burst-stimulating protocol (10 Hz for 3 seconds, repeated every 4 seconds), ChR2-induced synaptic release suppressed spontaneous action potential firing in arcuate NPY neurons (Figure 1, G and H, and Supplemental Figure 1A; supplemental material available online with this article; https://doi.org/10.1172/JCI130340DS1). These findings indicated a specific inhibitory synaptic connection between GABAergic aBNST neurons and orexigenic AgRP/NPY neurons but not anorexigenic POMC neurons in the arcuate nucleus.

### Optogenetic stimulation of GABAergic aBNST fibers in the arcuate nucleus suppresses feeding.

To determine whether GABAergic aBNST projections to arcuate NPY neurons regulate feeding, Cre-dependent ChR2-mCherry– or YFP-containing AAVs were injected into the aBNST of 4-month-old male Vgat-Cre mice and their WT littermates, with optical fibers positioned above or within the arcuate nucleus (Figure 1I and Supplemental Figure 1B). Two weeks after surgery, overnight-fasted mice were photostimulated using the same burst protocol as applied in the ex vivo slice studies. Stimulation of ChR2-mCherry–expressing aBNST to arcuate nucleus efferent fibers significantly suppressed food intake after 4 hours, an effect that persisted following cessation of the light stimulus (Figure 1, J and K). This was not observed in photostimulated WT mice injected with ChR2-mCherry (Supplemental Figure 1C) or in YFP-injected Vgat-Cre mice (Supplemental Figure 1D). Stimulation of BNST fibers at a more dorsal site (~0.9 mm from the arcuate nucleus) did not alter feeding in ChR2-mCherry–injected WT or Vgat-Cre mice, suggesting that the effect was specific to the arcuate nucleus (Supplemental Figure 1, E and F).

### A subset of GABAergic aBNST to arcuate nucleus projection neurons express nociceptin and inhibit NPY neurons.

The aBNST displays neurochemical heterogeneity, but recent evidence has shown that expression of specific neuropeptides may underlie the functional specificity of BNST GABAergic neuron populations (23). We therefore sought to define whether a specific subpopulation of GABAergic neurons was connected to arcuate AgRP/NPY neurons and modulated feeding. A number of peptides expressed in BNST neurons are implicated in feeding and are also involved in stress and anxiety, but we focused our initial studies on 2 opioid peptides: dynorphin and nociceptin. Dynorphin has previously been shown to inhibit NPY neuron excitability in slice recordings, and activation of DMH dynorphin neurons in vivo inhibits AgRP neurons to suppress food intake (7, 24). Arcuate AgRP/NPY neurons express the receptor for nociceptin (called the NOP receptor), and exogenous nociceptin inhibits arcuate neurons by activation of a resting K^+^ conductance (25, 26). To explore a potential connection between aBNST dynorphin neurons and AgRP/NPY neurons, we crossed mice expressing Cre recombinase driven by the dynorphin promoter (*Pdyn*-Cre) with *Npy*-hrGFP mice and injected ChR2-mCherry AAVs into the aBNST. In agreement with previous studies (27), we observed somas expressing mCherry in the aBNST and the presence of axons in the arcuate nucleus (data not shown). However, photoexcitation of aBNST dynorphin axons failed to elicit a synaptic response in arcuate NPY neurons [*n* = 15 neurons from 5 mice, paired *t* test, *t* (14) = 1.09, *P* = 0.30], indicating no functional connection between these neuron populations.

**Figure 2:**
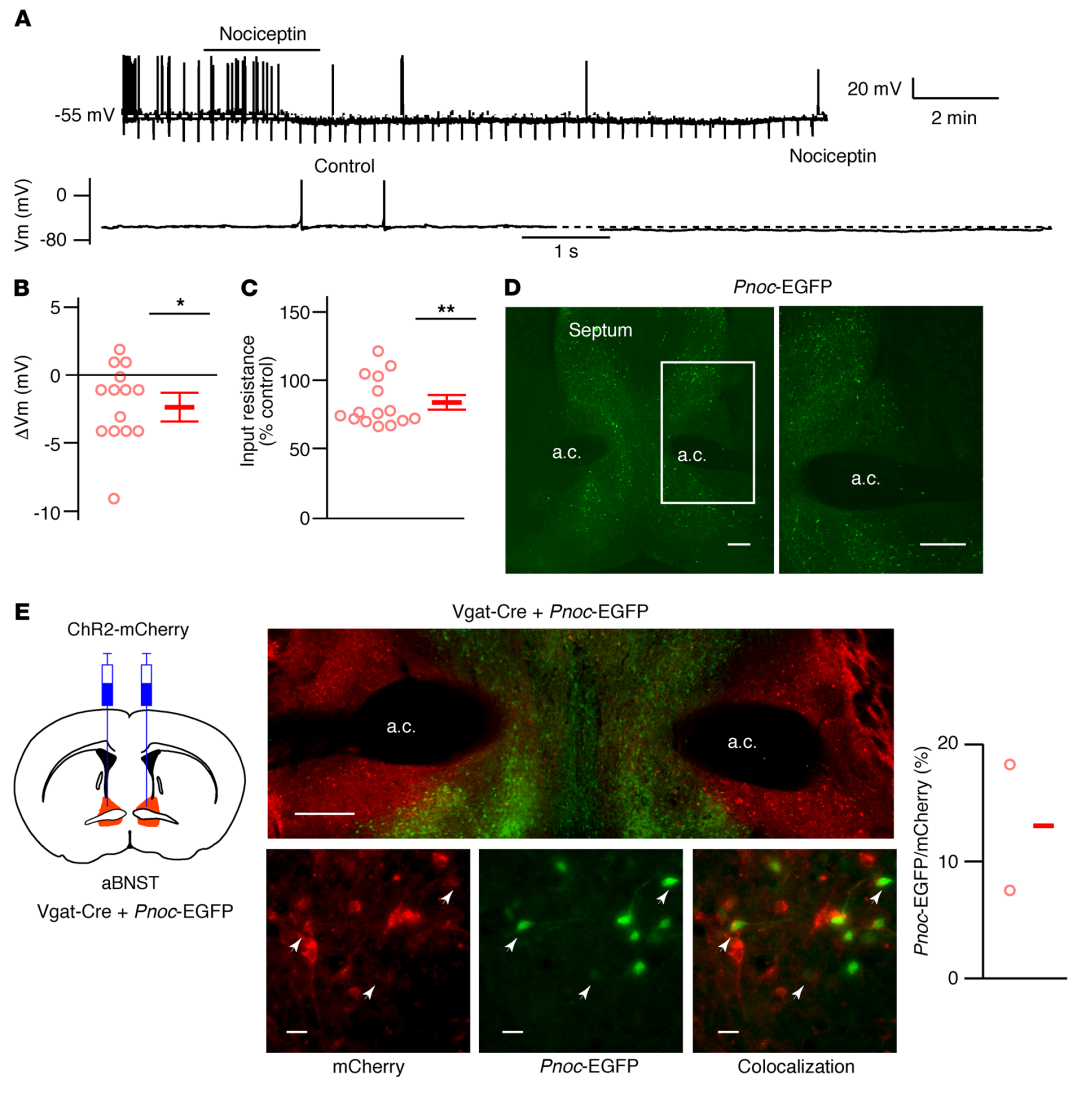
Nociceptin is expressed in a subpopulation of Vgat neurons. (**A**) Voltage traces (expanded below) from a NPY neuron during 0.5 μM nociceptin application, where indicated (*n* = 15). (**B** and **C**) Nociceptin-induced change in membrane potential (ΔVm) (**B**) and input resistance (**C**) in NPY neurons. Data represent the mean ± SEM. *n* = 15 neurons from 8 mice. *P < 0.05, Vm: 1-sampled *t* test, *t* (14) = 2.88, *P* = 0.012; **P < 0.01, input resistance: 1-sample *t* test, *t* (14) = 4.05, *P* = 0.0012. (**D**) Image of a coronal section (enlarged inset on the right) containing EGFP-expressing neurons driven by the *Pnoc* promoter (*n* = 10). Scale bars: 200 μm. (**E**) Diagram of Vgat-Cre mice crossed with *Pnoc*-EGFP mice and injected with ChR2-mCherry AAV. Low-magnification mosaic (top middle) and expanded (bottom middle) images showing *Pnoc*-EGFP (green) expression in a subpopulation of Vgat-Cre neurons expressing ChR2-mCherry (red). Arrowheads indicate colocalization of mCherry and *Pnoc*-EGFp. Scale bars: 200 μm (low-magnification images) and 20 μm (high-magnification images). Graph shows the percentage of ChR2-mCherry–expressing Vgat neurons that coexpressed *Pnoc*-EGFP. The mean from 2 mice is shown (338 of 1103 Vgat neurons coexpressed *Pnoc*-EGFP).

We then explored the effect of nociceptin on AgRP/NPY neuron excitability. Although the inhibitory role of nociceptin on arcuate POMC and gonadotrophin-releasing hormone neurons in electrophysiology studies is documented (26), the actions of nociceptin on AgRP/NPY excitability are less clear. In ex vivo electrophysiology studies, we observed a nociceptin-induced hyperpolarization in arcuate NPY neurons that was associated with a decrease in input resistance consistent with the activation of a cell membrane conductance (Figure 2, A–C). These data therefore suggested the possibility that nociceptin, released from aBNST neurons (that perhaps coexpress GABA), inhibits arcuate AgRP/NPY neurons to suppress feeding. However, nociceptin-expressing cells are also found in the ventromedial hypothalamus (VMH), and injection of nociceptin agonists to this brain region increases feeding (28, 29). Therefore, we next investigated whether VMH neurons, which may express and release endogenous nociceptin in the arcuate nucleus, directly synapse with arcuate NPY neurons. We injected Cre-dependent ChR2-mCherry AAVs into the VMH of mice expressing Cre recombinase driven by the promoter of steroidogenic factor 1 (Sf1-Cre), which captures the vast majority of VMH neurons, and performed ex vivo ChR2-assisted circuit mapping. Photostimulation of Sf1 axons produced a robust glutamatergic input to arcuate POMC neurons, but this was absent in NPY neurons (Supplemental Figure 2, A–C, and data not shown). This suggests that VMH glutamatergic neurons, including those that may express nociceptin, do not directly synapse with AgRP/NPY neurons to control their activity and hence feeding.

To determine whether nociceptin-expressing aBNST neurons synapse with arcuate AgRP/NPY neurons, we used a bacterial artificial chromosome–transgenic (BAC-transgenic) mouse line expressing EGFP under transcriptional control of the prepronociceptin (*Pnoc*) promoter (*Pnoc*-EGFP). We detected EGFP expression in the aBNST and in other regions such as the septum and preoptic area (Figure 2D), consistent with the Allen Brain Atlas (30), our in situ data (Supplemental Figure 3A), and the work of others (31–33). Immunohistochemical analysis demonstrated that GFP-labeled neurons coexpressed nociceptin in the aBNST (Supplemental Figure 3B). To determine whether *Pnoc*-EGFP neurons in the aBNST colocalized with Vgat-Cre neurons, we crossed the *Pnoc*-EGFP and Vgat-Cre mice and injected Cre-dependent ChR2-mCherry AAVs into the aBNST. We found that approximately 13% of Vgat-Cre neurons expressing mCherry colocalized with *Pnoc*-EGFP in the aBNST (Figure 2E). Together, these findings confirmed the utility of using the *Pnoc*-EGFP mouse to explore the role of aBNST nociceptin neurocircuitry in the regulation of AgRP/NPY neurons.

**Figure 3:**
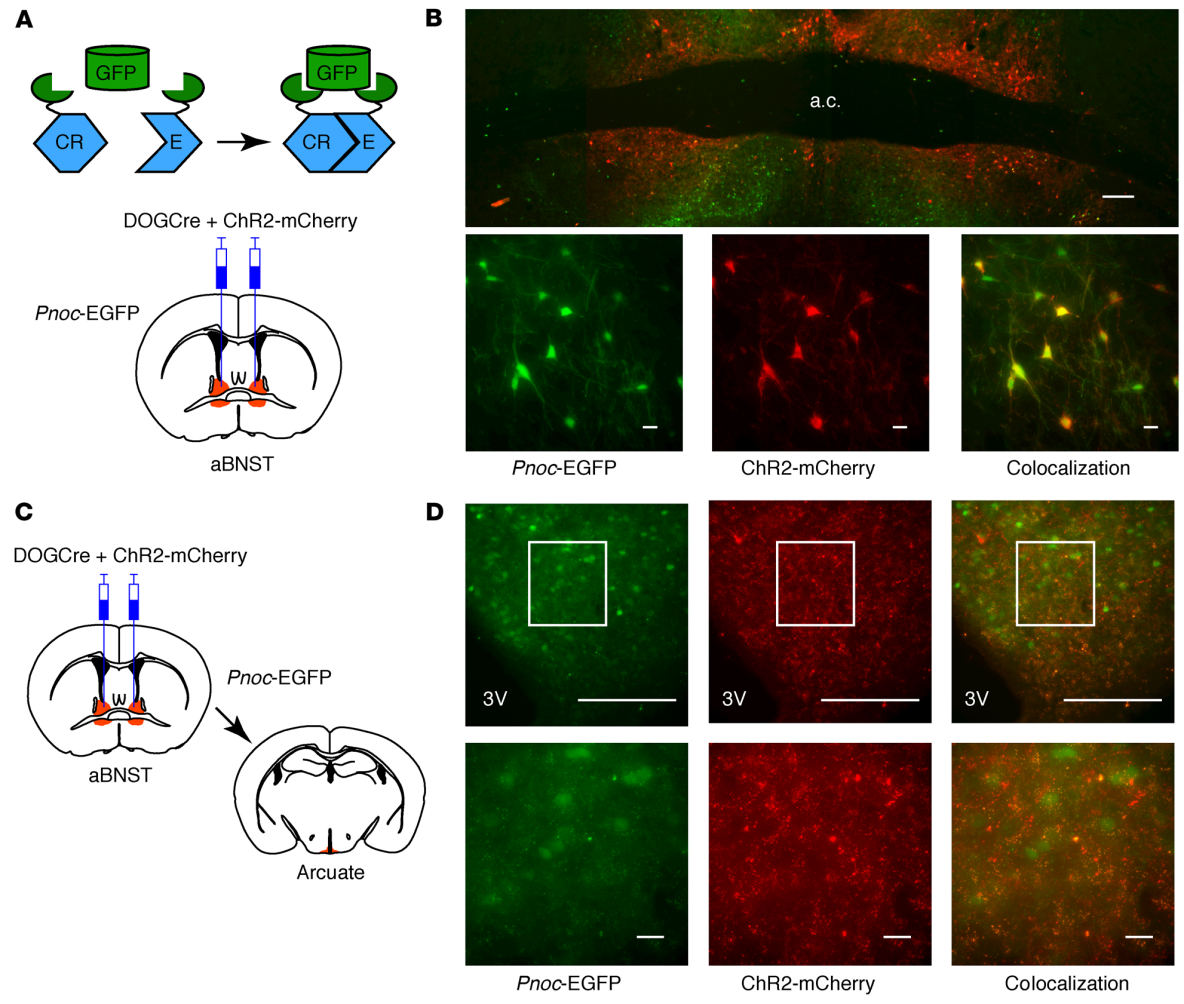
Nociceptin neurons project to the arcuate nucleus. (**A**) Diagrams of the DOGCre technology used to express Cre recombinase in *Pnoc*-EGFP neurons (top) and injection of DOGCre (C-CreintG and N-CretrcintG) and ChR2-mCherry AAVs into the aBNST (bottom). (**B**) Mosaic images (enlarged images shown below) showing the expression of *Pnoc*-EGFP (green) and ChR2-mCherry (red) in the aBNST (*n* = 47). (**C**) Diagrams showing the injection of DOGCre and ChR2-mCherry AAVs into the aBNST and the corresponding arcuate nucleus section. (**D**) Coronal sections (enlarged insets shown below) showing ChR2-mCherry expression in axons in the arcuate nucleus (red) and EGFP driven by the *Pnoc* promoter (green). *n* = 10. Scale bars: 200 μm (low-magnification images) and 20 μm (high-magnification images).

**Figure 4:**
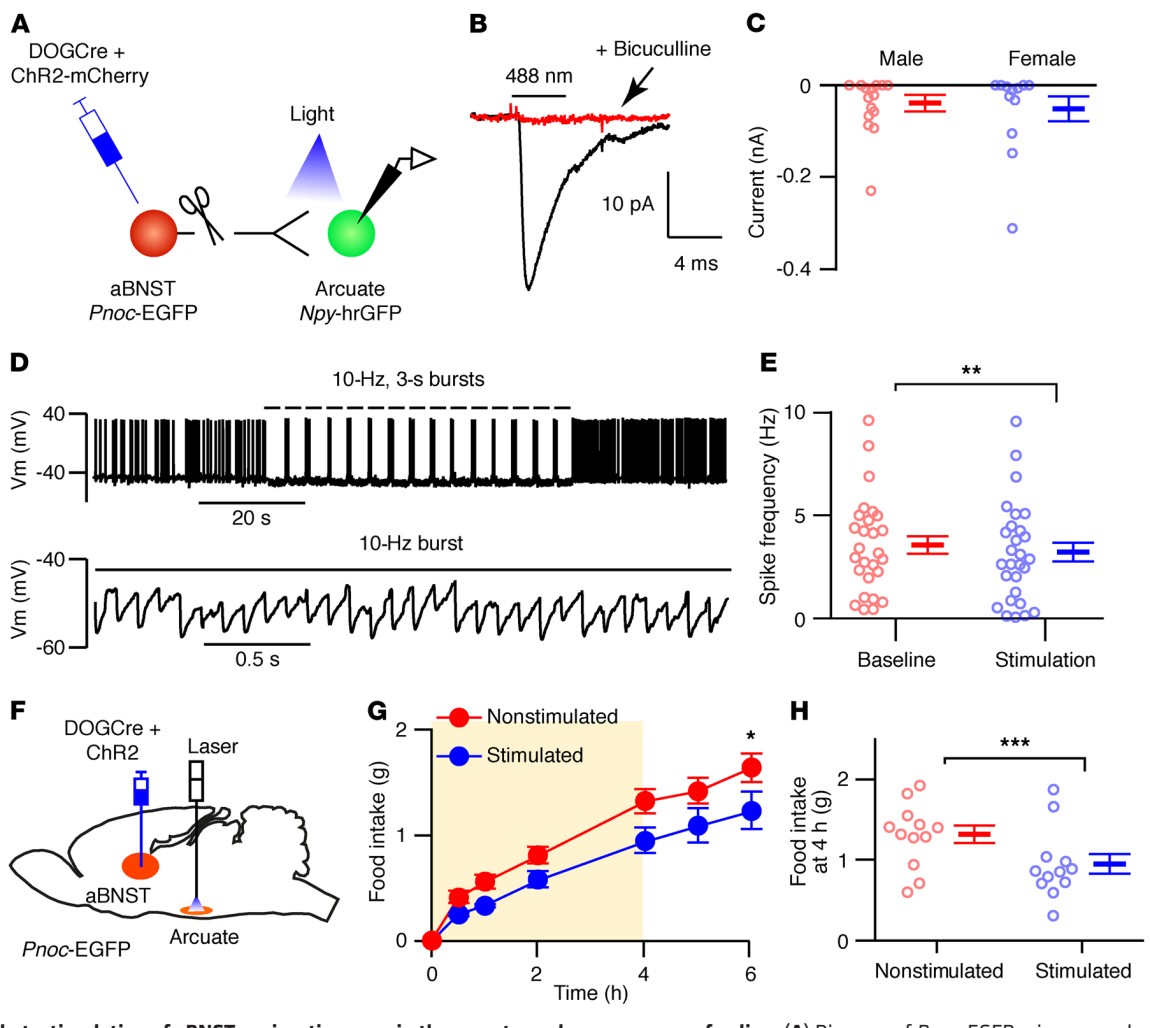
Photostimulation of aBNST nociceptin axons in the arcuate nucleus suppresses feeding. (**A**) Diagram of *Pnoc*-EGFP mice crossed with *Npy*-hrGFP mice and injected with DOGCre and ChR2-mCherry AAVs into the aBNST. (**B**) Ensembled synaptic currents from an arcuate NPY neuron (*n* = 27) in the absence (black) and presence (red) of 20 μM bicuculline. (**C**) Photostimulated synaptic currents in arcuate NPY neurons from male (*n* = 15 neurons from 5 mice) and female (*n* = 12 neurons from 3 mice) mice. Data represent the mean ± SEM. An unpaired *t* test was performed for comparison between sexes [*t* (25) = 0.35, *P* = 0.73]. (**D**) Voltage traces (expanded below) from an arcuate NPY neuron during photostimulation (where indicated) of ChR2-mCherry–expressing aBNST axons from *Pnoc*-EGFP mice (*n* = 28). (**E**) Arcuate NPY neuronal action potential frequency (*n* = 28 neurons from 9 mice) before (baseline) and during photostimulation of aBNST axons. Data represent the mean ± SEM. **P < 0.01, by paired *t* test, *t* (27) = 3.33, *P* = 0.003. (**F**) Diagram illustrating a sagittal section containing the aBNST injected with DOGCre and ChR2-mCherry AAVs and the arcuate nucleus implanted with optical fibers. (**G**) Cumulative food intake following an overnight fast of *Pnoc*-EGFP mice injected with DOGCre and ChR2-mCherry AAVs into the aBNST during (shaded area; 3-second, 10-Hz bursts every 4 seconds) and after photostimulation of aBNST *Pnoc* fibers in the arcuate nucleus. Food intake was measured without (Nonstimulated, red) and with photoexcitation (Stimulated, blue). Data represent the mean ± SEM. *n* = 12 mice. Two-way repeated-measures ANOVA [interaction: *f* (6,132) = 2.15, *P* = 0.052; stimulation: *f* (1,22) = 4.76, *P* = 0.040]. *P < 0.05, by Sidak’s post hoc test. (**H**) Food intake following the 4-hour photostimulation period for nonstimulated (red) and stimulated (blue) mice. Data represent the mean ± SEM. *n* = 12 mice. ***P < 0.001, by paired *t* test, *t* (11) = 4.52, *P* = 0.0009.

In order to target *Pnoc*-EGFP neurons with Cre-inducible AAV, we used the dependent-on-GFP (DOGCre) method, in which GFP-binding proteins are used to allow the molecular assembly of Cre recombinase on a GFP scaffold, thus permitting Cre-mediated recombination in cells that express GFP (Figure 3A and refs. 34, 35). A mix of AAVs containing the DOGCre viruses (C-CreintG and N-CretrcintG) and Cre-dependent ChR2-mCherry was injected into the aBNST of *Pnoc*-EGFP mice. Expression of mCherry in somas was restricted to the aBNST and colocalized completely with GFP-expressing neurons (Figure 3B). Ex vivo photostimulation of ChR2-mCherry–expressing aBNST neurons induced action potential firing in these cells (Supplemental Figure 4A), and axons expressing mCherry were observed in the arcuate nucleus, but to a lesser extent than that seen in the Vgat-Cre mouse studies (Figure 3, C and D). To determine whether these nerve fibers made synaptic connections with arcuate AgRP/NPY neurons, we crossed *Pnoc*-EGFP mice with *Npy*-hrGFP mice and injected DOGCre and ChR2-mCherry or synaptophysin-mCherry AAVs into the aBNST, taking advantage of the fact that the DOGCre technology does not recognize *Renilla*-GFP and thus would not lead to Cre-dependent ChR2-mCherry expression in the arcuate nucleus of *Npy*-hrGFP animals (Supplemental Figure 4, B and C). Synaptophysin-tagged mCherry clustered at the somas of arcuate *Npy*-hrGFP neurons and elsewhere in the mediobasal hypothalamus (Supplemental Figure 4, C and D). In slice recordings, photostimulation of aBNST nociceptin projections induced an inward current in arcuate *Npy*-hrGFP neurons from both male and female mice (Figure 4, A–C). The lack of sex differences with respect to synaptic connectivity suggests that this pathway is not sexually dimorphic. Moreover, when tested, these synaptic currents were blocked by a GABA_A_ receptor antagonist [*n* = 6 neurons from 3 mice, paired *t* test, *t* (5) = 3.21, *P* = 0.024], confirming the GABAergic nature of these neurons. Furthermore, using a burst pattern of light stimulation, photoexcitation of ChR2-mCherry–containing *Pnoc* fibers from the aBNST reduced the spontaneous action potential firing rate in arcuate *Npy*-hrGFP neurons (Figure 4, D and E, and Supplemental Figure 4E).

For a molecular characterization of BNST *Pnoc* neurons, we used the RiboTag method to enrich RNA from these cells (36, 37), subjected the RNA to RNA-Seq, and compared this expression profile to that of the whole aBNST. As shown by the volcano plot (Supplemental Figure 5 and Gene Expression Omnibus [GEO] database accession number GSE135982), 705 genes were enriched and a further 1762 genes were depleted in the aBNST of *Pnoc*-EGFP mice. Prepronociceptin was enriched by 4.97-fold (*P* = 1.91 × 10^–15^), confirming the selective targeting to *Pnoc* neurons. However, the relative expression levels of several other neuropeptides that are expressed in the BNST (e.g., corticotropin-releasing hormone, *Npy*, somatostatin) were not different. In contrast, the expression of preproenkephhalin (*Penk*) and prodynorphin (*Pdyn*) was depleted (5.33-fold, *P* = 4.45 × 10^–33^ and 5.59-fold, *P* = 0.01, respectively), consistent with our ChR2 mapping data that failed to demonstrate a connection between aBNST dynorphin axons and arcuate NPY neurons. Taken together, these studies suggest that aBNST nociceptin neurons represent a molecularly distinct GABAergic subpopulation synaptically connected to AgRP/NPY neurons with the potential to regulate feeding behavior.

### Optogenetic stimulation of Pnoc fibers from the aBNST suppresses feeding but does not elicit anxiety.

We next determined whether excitation of *Pnoc* fibers from the aBNST could suppress feeding. We injected ChR2-mCherry and DOGCre AAVs into the aBNST of 4-month-old male *Pnoc*-EGFP and WT littermate mice and positioned optical fibers within or above the arcuate nucleus (Figure 4F and Supplemental Figure 6A). Photostimulation significantly suppressed feeding after 4 hours in overnight-fasted and habituated *Pnoc*-EGFP mice (Figure 4, G and H) but had no effect on WT mice (Supplemental Figure 6B). In contrast, photostimulation of *Pnoc* nerve axons in dorsal regions (~0.6 mm) from the arcuate nucleus did not alter feeding in WT (Supplemental Figure 6C) or *Pnoc*-EGFP (Supplemental Figure 6D) ChR2-mCherry–injected mice. Given the proximity of the septum to the aBNST, the presence of *Pnoc*-EGFP neurons in this region and studies implicating septal projections in the suppression of feeding (38–40), we injected DOGCre with ChR2-mCherry AAVs into the septum of *Pnoc*-EGFP mice and placed optical fibers in the arcuate nucleus (Supplemental Figure 6E). However, we found that photostimulation of the arcuate nucleus projections did not alter feeding (Supplemental Figure 6F), suggesting that nociceptin-expressing septal neurons that may project to the arcuate nucleus do not regulate food intake.

**Figure 5:**
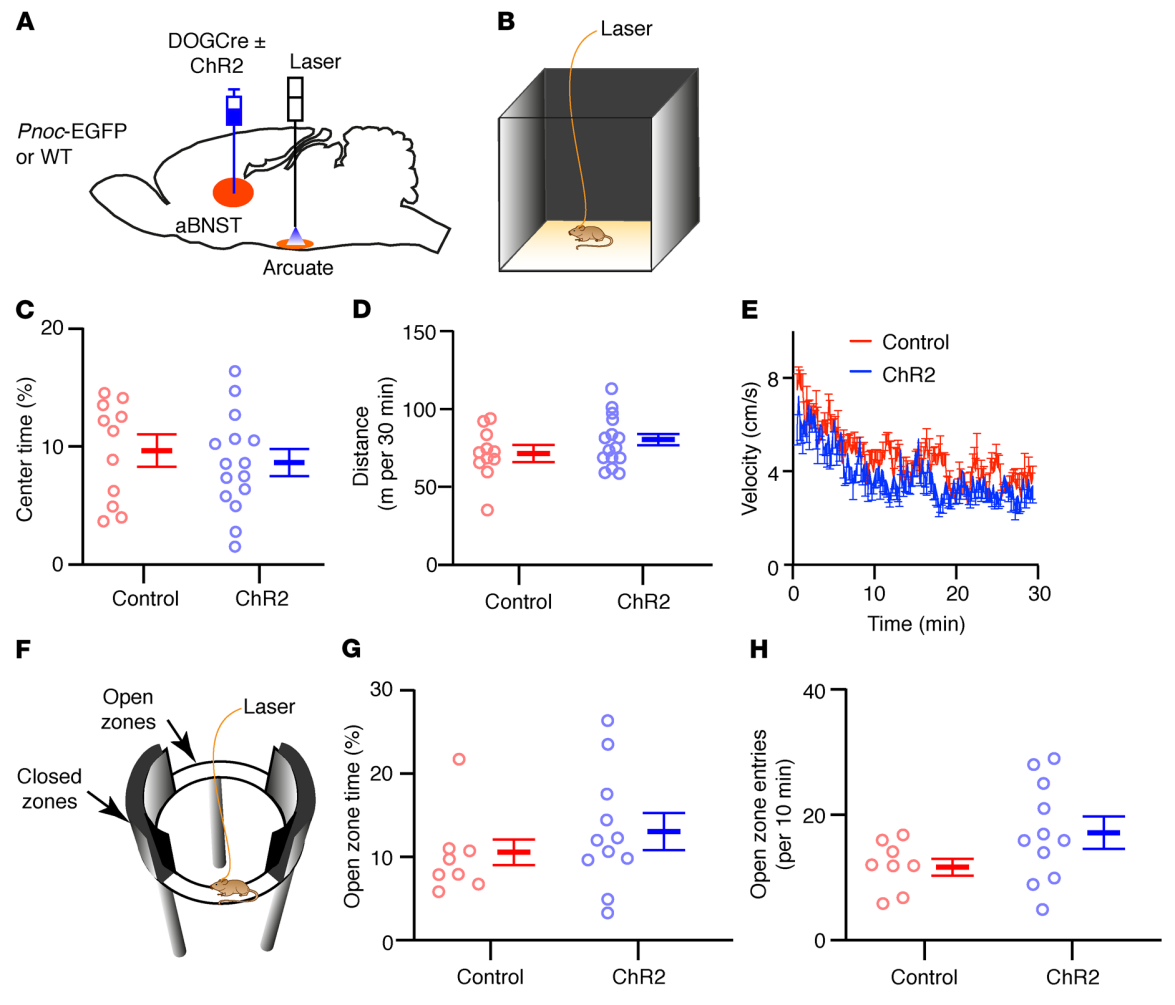
Optogenetic stimulation of aBNST nociceptin fibers in the arcuate nucleus does not induce anxiety-like behavior. (**A**) Diagram illustrating the injection of *Pnoc*-EGFP mice and WT littermate controls with DOGCre, with or without ChR2-mCherry AAVs, into the aBNST and implantation of optical fibers into the arcuate nucleus. (**B**) Schematic representation of mice tethered to a 470-nm laser and photostimulated (3-second, 10-Hz bursts every 4 seconds) for 30 minutes in a novel open-field arena. (**C**–**E**) Percentage of time spent in the center (**C**) [unpaired *t* test, *t* (24) = 0.63, *P* = 0.53], total distance traveled (**D**) [unpaired *t* test, *t* (24) = 1.20, *P* = 0.24], and mouse velocity (**E**) [2-way repeated-measures ANOVA, interaction: *f* (173,4152) = 1.08, *P* = 0.241; ChR2 expression: *f* (1,24) = 1.44, *P* = 0.241] in the open-field arena for mice expressing (blue, *n* = 15 mice) or not expressing (red, *n* = 11 mice) ChR2-mCherry. Data represent the mean ± SEM. (**F**) Schematic representation of mice tethered to a 470-nm laser and photostimulated (3-second, 10-Hz bursts every 4 seconds) for 10 minutes on a novel elevated zero maze. (**G**) Percentage of time spent in the anxiogenic open zones [unpaired *t* test, *t* (17) = 0.99, *P* = 0.33] and (**H**) number of entries into the anxiogenic open zones [unpaired *t* test, *t* (17) = 1.74, *P* = 0.10] for mice expressing (blue, *n* = 11 mice) or not expressing (red, *n* = 8 mice) ChR2-mCherry. Data represent the mean ± SEM.

In view of the role of the aBNST in anxiety-related behaviors, antidromic optogenetic stimulation could alter feeding by changing the affective state of the mouse. To exclude this possibility, we photostimulated ChR2-mCherry–expressing aBNST to arcuate nucleus *Pnoc* axons in 4-month-old male *Pnoc*-EGFP or WT littermate mice in a novel open-field arena (Figure 5, A–E) and in an elevated zero maze (Figure 5, F–H). Mice were continuously photostimulated using the burst protocol for 30 minutes in the open-field task and for 10 minutes on the elevated zero maze. We observed no differences in the avoidance of the anxiogenic center of the open-field arena, total distance covered, or velocity when compared with mice that only expressed DOGCre or were WT (Figure 5, C–E). Similarly, time spent in and entrances to the anxiogenic open zones of the elevated zero maze were not different between ChR2-mCherry–expressing mice and littermate controls (Figure 5, G and H). These tests suggest that the reduction in feeding is unlikely to be due to an overt anxiety-related behavior.

### aBNST Pnoc neurons increase activity during feeding.

**Figure 6:**
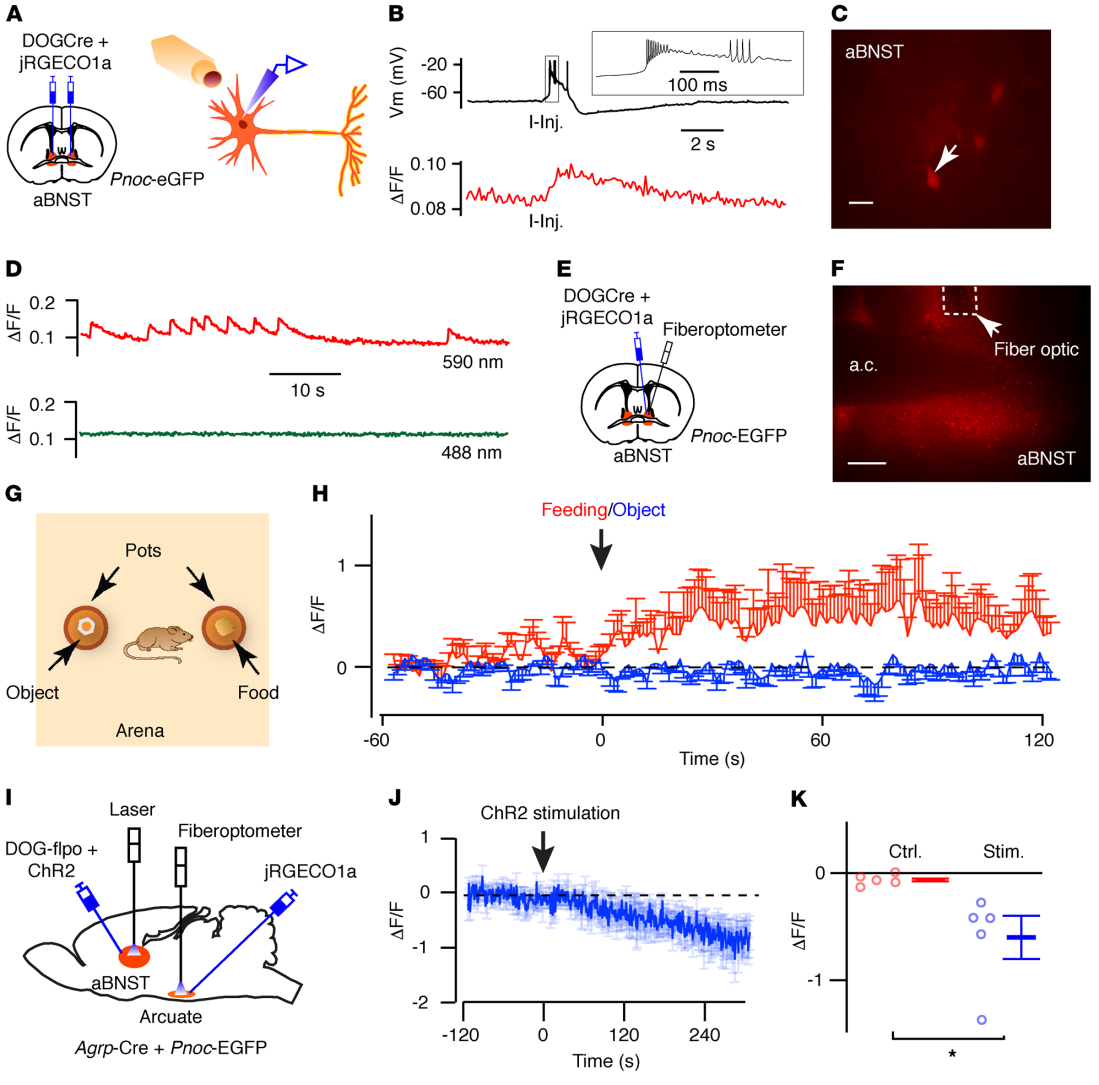
Activity of aBNST nociceptin neurons increases during the initiation of feeding. (**A**) Diagram illustrating injection of DOGCre and jRGECO1a AAVs into the aBNST of *Pnoc*-EGFP mice for ex vivo imaging and recording. (**B**) Simultaneous measurements of electrical excitability (Vm, top) and fluorescence intensity (ΔF/F, bottom) in aBNST jRGECO1a–expressing neurons (*n* = 3). Action potential firing was evoked by depolarizing current injections (I-Inj.) and correlated with increased fluorescence. (**C** and **D**) Image of aBNST neuron (**C**) and corresponding changes in spontaneous jRGECO1a fluorescence (ΔF/F) (**D**) at 590 nm but not 488 nm (*n* = 5). (**E**) Diagram illustrating injection of DOGCre and jRGECO1a AAVs into the aBNST of *Pnoc*-EGFP mice, with implantation of optical fibers into the aBNST. (**F**) jRGECO1a expression in the aBNST and optical fiber location. (**G**) Illustration of the open-field arena containing 2 pots (with a novel object or food). (**H**) Fluorescence intensity (ΔF/F) in mice approaching a novel object (blue) or initiating feeding (red). Data represent the mean ± SEM. *n* = 7 mice. Two-way repeated-measures ANOVA [interaction: *f* (239,2868) = 1.44, *P* < 0.0001; time: *f* (239,2868) = 1.35, *P* < 0.001; feeding versus object: *f* (1,12) = 2.70, *P* = 0.14]. (**I**) Diagram of intercrossed *Pnoc*-EGFP and *Agrp*-Cre mice injected with DOG-flpo and flp-dependent ChR2-mCherry AAVs into the aBNST and Cre-dependent jRGECO1a AAV into the arcuate nucleus. Optical fibers were placed in the aBNST for ChR2 stimulation and in the arcuate nucleus for jRGECO1a activity recording. (**J**) Fluorescence intensity (ΔF/F) in AgRP neurons before and after aBNST photostimulation, where indicated. Data indicate the mean ± SEM. *n* = 5 mice. (**K**) AgRP activity (ΔF/F) for mice shown in **J** before (Ctrl.) and after stimulation (Stim.). Data represent the mean ± SEM. *P < 0.05, by paired *t* test [*t* (4) = 2.97, *P* = 0.041]. Scale bars: 20 μm (**C**) and 200 μm (**F**).

Next, we used fiber photometry to assay endogenous activity of aBNST *Pnoc* neurons in response to food presentation. Given that *Pnoc*-driven EGFP expression would interfere with GFP-based calcium indicators (e.g., GcAMP6), we examined calcium dynamics using a red fluorescent calcium indicator (jRGECO1a) (41). The aBNST of *Pnoc*-EGFP mice was injected with AAVs expressing DOGCre and a Cre-dependent jRGECO1a construct (Figure 6A). In ex vivo electrophysiological studies, red fluorescence intensity dynamically increased in response to action potential firing induced by current injections (Figure 6B). Furthermore, we observed spontaneous changes in activity when the calcium indicator was excited with a 590-nm but not a 488-nm light source (Figure 6, C and D), suggesting that endogenous EGFP expression from the *Pnoc* locus does not itself manifest as a dynamic change in fluorescence. We next implanted optical fibers into the mediodorsal region of the aBNST (Figure 6, E and F), in view of its reported connectivity with the arcuate nucleus (16). jRGECO1a fluorescence was stimulated and detected using a modified fiberoptometer. We sequentially exposed fasted mice to a novel object placed in 1 pot, followed 10 minutes later by exposure to chow in a second pot (Figure 6G). Fluorescence (ΔF/F) intensity increased at the initiation of feeding but did not change when the mice approached the novel object (Figure 6H), indicating a specific response of aBNST *Pnoc* neurons to food and suggesting that they are sensitive to nutritional cues. To confirm that optogenetic stimulation of aBNST *Pnoc* neurons inhibited arcuate AgRP neurons in vivo, we intercrossed *Agrp*-Cre mice with *Pnoc*-EGFP mice and expressed the jRGECO1a reporter in AgRP neurons and ChR2 in aBNST *Pnoc*-EGFP neurons. To exclude the potential expression of ChR2 in AgRP neurons, we injected a DOG AAV expressing flp recombinase (DOG-flpo) and an flp-dependent, rather than Cre-dependent, ChR2-mCherry (fDIO-ChR2-mCherry) AAV into the aBNST (Figure 6I). Expression of mCherry in the aBNST was colocalized with EGFP-expressing neurons, and jRGECO1a expression was observed in the arcuate nucleus (Supplemental Figure 7, A and B). We positioned optical fibers in the aBNST to photostimulate ChR2 *Pnoc*-EGFP neurons and in the arcuate nucleus to record calcium activity in AgRP neurons (Figure 6I). Mice were habituated to tethering and an open-field arena, and then ad libitum–fed mice were photostimulated for 2 minutes using the 10-Hz burst stimulation protocol. Consistent with our ex vivo data, excitation of aBNST *Pnoc* neurons reduced AgRP activity in vivo (Figure 6, J and K).

### Chronic ablation of aBNST Pnoc-EGFP neurons increases body weight.

**Figure 7:**
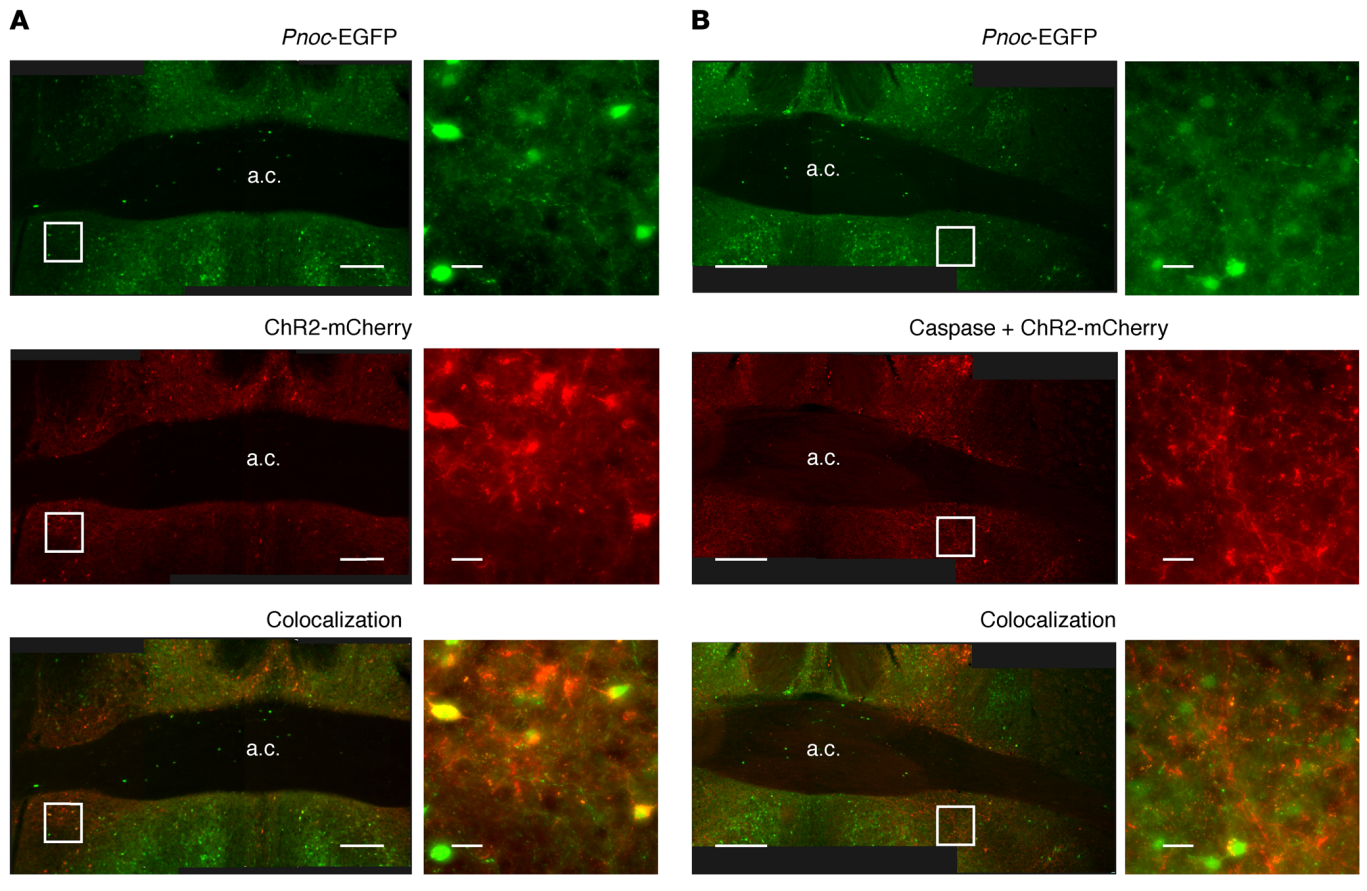
Caspase-mediated ablation of aBNST nociceptin neurons. (**A** and **B**) Low-magnification mosaic images (left) and enlarged insets (right) for mice not expressing (**A**, *n* = 4) or expressing (**B**, *n* = 7) caspase 3 in aBNST *Pnoc* neurons. Images show EGFP expression driven by the *Pnoc* promoter (top panels), Cre-dependent expression of ChR2-mCherry (middle panels), and colocalization (bottom panels). Scale bars: 200 μm (low-magnification images) and 20 μm (high-magnification images).

**Figure 8:**
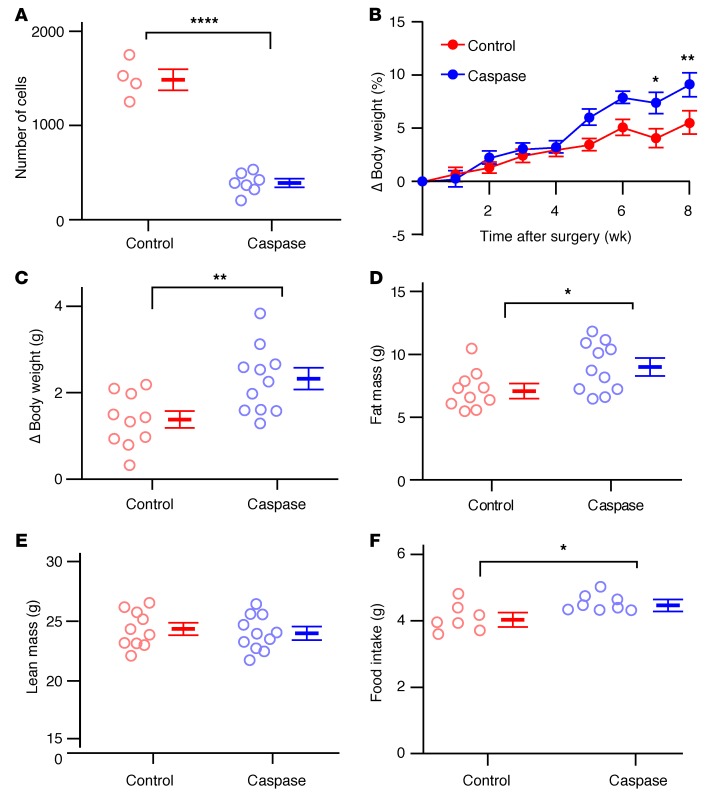
Loss of aBNST *Pnoc* neurons increases adiposity and body weight. (**A**) Number of mCherry-expressing aBNST somas in control (red, *n* = 4) and caspase 3–treated (blue, *n* = 7) mice. Data represent the mean ± SEM. ****P < 0.0001, by unpaired *t* test [*t* (9) = 11.88, *P* = 4.19 × 10^–7^]. (**B**) Cumulative change in body weight (percentage of pre-surgery weight) in control (red, *n* = 10) and caspase 3–treated (blue, *n* = 11) mice. Data represent the mean ± SEM. Two-way repeated-measures ANOVA [interaction: *f* (8,152) = 3.37, *P* = 0.0014; control versus caspase treatment: *f* (1,19) = 5.02, *P* = 0.037]. *P < 0.05 and **P < 0.001, by Sidak’s post hoc test. (**C**) Change in body weight 6 weeks after surgery in control (red, *n* = 10) and caspase 3–treated (blue, *n* = 11) mice. Data represent the mean ± SEM. **P < 0.01, by unpaired *t* test [*t* (19) = 3.07, *P* = 0.0063]. (**D**) Fat mass [unpaired *t* test, *t* (19) = 2.43, *P* = 0.025] (*P < 0.05) and (**E**) lean mass [unpaired *t* test, *t* (19) = 0.48, *P* = 0.63] six weeks after surgery in control mice (red, *n* = 10) and caspase 3–treated mice (blue, *n* = 11). Data represent the mean ± SEM. (**F**) Ad libitum food intake in control (red, *n* = 7) and caspase 3–treated (blue, *n* = 8) mice. Data represent the mean ± SEM. *P < 0.05, by unpaired *t* test [*t* (13) = 2.53, *P* = 0.025].

To gain insights into the long-term physiological effects on energy homeostasis following disruption of aBNST *Pnoc* neuron function, we performed caspase-mediated ablation of these neurons (42). We injected the aBNST of 5-month-old *Pnoc*-EGFP male mice with DOGCre and ChR2-mCherry AAVs with and without a Cre-dependent caspase 3 AAV to induce cell death. Postmortem analysis (~10 weeks after injection) confirmed the expression of ChR2-mCherry and, by inference, Cre recombinase expression (Figure 7, A and B). Although we observed mCherry fibers in the aBNST in both control (Figure 7A) and caspase 3–expressing (Figure 7B) mice, the number of intact somas was reduced by approximately 75% in the caspase 3–treated mice, confirming a significant loss of aBNST *Pnoc* neurons (Figure 8A). In the ablated mice, consistent with the time taken for virus expression and cell death, body weights increased, diverging from the body weights of control animals 4 weeks after virus injections (Figure 8, B and C). Increased body weight was associated with adiposity (Figure 8D), but we observed no differences in lean body mass (Figure 8E). Furthermore, caspase-treated mice ate more than did littermate controls in ad libitum feeding conditions (Figure 8F). These findings indicate a physiologically relevant role for the aBNST *Pnoc*–to–AgRP/NPY neuronal circuitry, as loss of the inhibitory effects of this pathway on AgRP/NPY would be anticipated to lead to increased activity of these neurons.

### Nociceptin neurons in the aBNST provide a gateway from “higher centers.”

**Figure 9:**
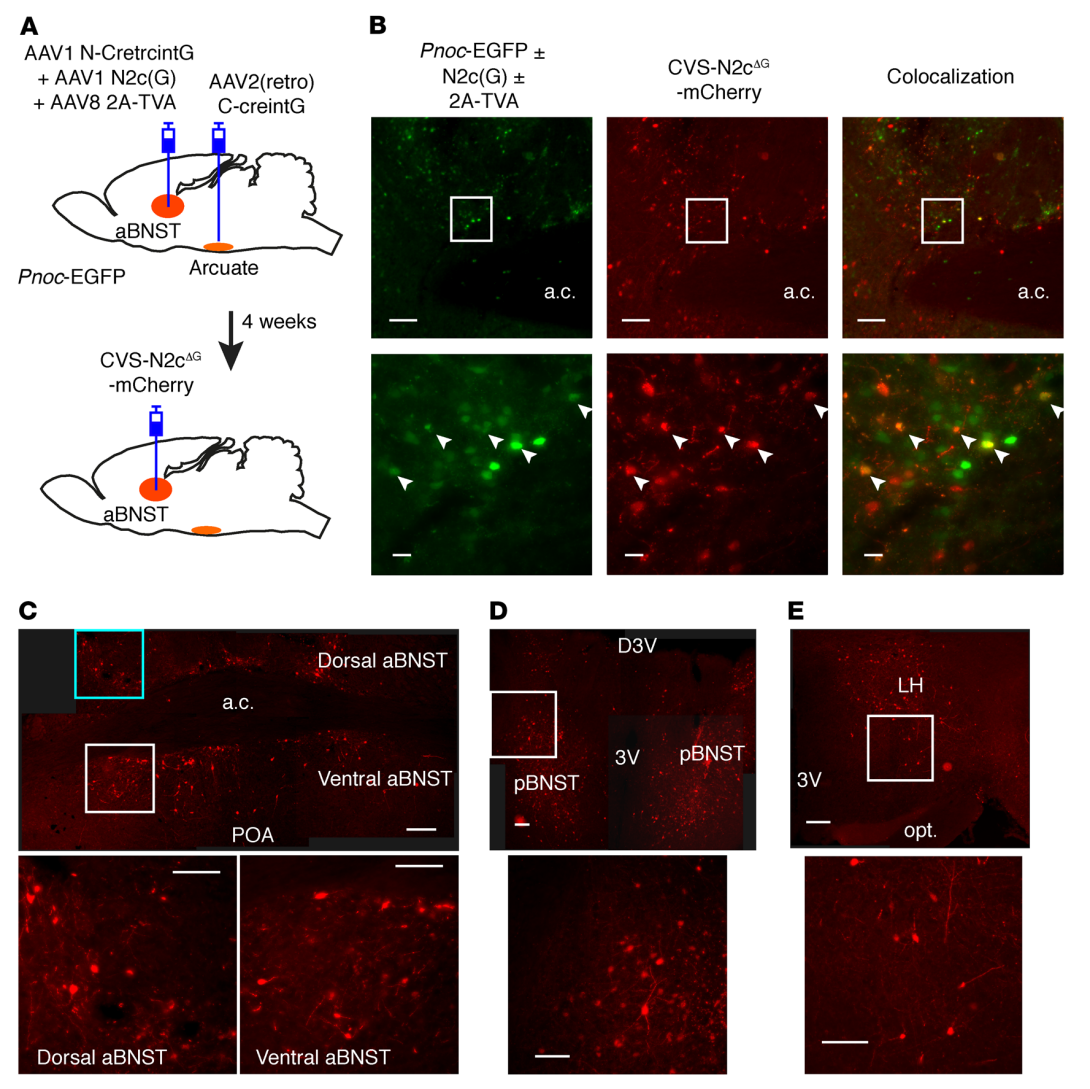
A population of aBNST *Pnoc* neurons receives inputs from the hypothalamus. (**A**) Diagram of injection of C-CreintG [serotyped with AAV2(retro)] into the arcuate nucleus and N-CretrcintG (serotyped with AAV1) into the aBNST of *Pnoc*-EGFP mice to drive Cre recombinase expression. A Cre-dependent avian retroviral receptor (2A-TVA) tagged with GFP and N2c-glycoprotein [N2c(G)] tagged with GFP were simultaneously injected into the aBNST. After 4 weeks to allow Cre-dependent expression, a glycoprotein-deficient rabies virus (CVS-N2c^ΔG^) expressing mCherry was injected into the aBNST. (**B**) Representative images (*n* = 3) of a coronal section containing EGFP-expressing neurons driven by the *Pnoc* promoter and/or the tagged avian receptor and glycoprotein (left). Expression of mCherry driven by the rabies virus (middle) colocalized (right) with a small number of GFP-positive neurons (as shown by the arrows). Scale bars: 200 μm (low-magnification images) and 20 μm (high-magnification images corresponding to the boxed regions in the upper panels). (**C**–**E**) Low-magnification mosaic images (top) and enlarged insets (bottom) showing the expression of mCherry (*n* = 3 mice) driven by the rabies virus in presynaptic neurons in the aBNST (**C**), pBNST (**D**), and LH (**E**)**.** Scale bars: 200 μm (low-magnification images) and 100 μm (high-magnification images). D3V, dorsal third ventricle; opt., optic tract.

The BNST acts as an integrative center, receiving inputs from homeostatic and nonhomeostatic higher brain centers. aBNST *Pnoc* neurons might therefore receive inputs from such areas relevant to feeding behavior. Thus, to map presynaptic inputs to aBNST *Pnoc* neurons from such brain regions, we used the glycoprotein-deleted rabies monosynaptic tracing strategy (43). To restrict our mapping to only the aBNST neurons that projected to the mediobasal hypothalamus, we took advantage of the absolute requirement for C-CreintG and N-CretrcintG to be expressed together to make Cre recombinase using the DOGCre approach (data not shown and ref. 34). We combined this with packaging of the C-CreintG construct using the AAV2(retro) serotype (44), which allows retrograde transport from distal axonal arbors such as the aBNST *Pnoc* cell population projecting to the arcuate nucleus. To test this approach, we initially injected AAV2(retro) C-CreintG into the arcuate nucleus and AAV1-serotyped N-CretrcintG and ChR2-mCherry into the aBNST of *Pnoc*-EGFP mice (Supplemental Figure 8A). This approach ensured that only neurons projecting to the arcuate nucleus that expressed all 3 viruses would express Cre recombinase and, hence, ChR2-mCherry. We observed a small number (~10) of fluorescent neurons in the aBNST (Supplemental Figure 8B) and reasoned that these hypothalamic projecting aBNST neurons could be used as a seeding population to investigate presynaptic inputs through monosynaptic rabies tracing. Therefore, we injected AAV2(retro) C-CreintG into the arcuate nucleus and AAV1 N-CretrcintG into the aBNST of *Pnoc*-EGFP mice to induce expression of Cre recombinase. We also injected the aBNST with AAV8-serotyped virus particles containing a Cre-dependent avian retroviral receptor (TVA, type-2A) tagged with GFP and AAV1-serotyped Cre-dependent N2c-glycoprotein [N2c(G)] with a nuclear marker (h2BG) and GFP (Figure 9A). After 4 weeks, we injected an EnvA-pseudotyped glycoprotein-deficient rabies virus (CVS-N2c^ΔG^) that expressed mCherry into the aBNST and allowed expression for 7 days prior to postmortem anatomical analysis (43). mCherry expression was observed throughout the aBNST (Figure 9, B and C) but only colocalized with a small number of GFP neurons that may have arisen from the *Pnoc*-EGFP and/or GFP-tagged N2c(G) and TVA constructs (Figure 9B). We also detected mCherry expression in the posterior BNST (pBNST) (Figure 9D), consistent with the high degree of synaptic connectivity within this nucleus. We next determined the magnitude of synaptic input by counting the number of mCherry-expressing somas that were present outside the BNST (mean of 3 mice, 1410 ± 423 cells per mouse). The hypothalamus accounted for two-thirds of the synaptic input to aBNST *Pnoc* neurons. We observed substantial inputs from the preoptic area (POA) (Figure 9C and Supplemental Figure 8, C and H), the lateral hypothalamus (LH) (Figure 9E and Supplemental Figure 8H), and the anterior hypothalamus (AH) (Supplemental Figure 8, D, E, and H). We also detected mCherry expression at the edges of the PVN (Supplemental Figure 8, E and H) and in several other hypothalamic regions such as the DMH (Supplemental Figure 8, F and H) and VMH (Supplemental Figure 8, G and H). Interestingly, the arcuate nucleus had very little mCherry expression, and this was mainly limited to the posterior division. Consistent with known BNST inputs (16, 22, 45–48), the lateral septum (Supplemental Figure 9, A and G) and the cingulate cortex (Supplemental Figure 9, B and G) were moderately connected. We detected lower levels of mCherry expression in the subiculum (Supplemental Figure 9, C and G), which is the major output region of the hippocampus, and in the retrosplenial cortex (Supplemental Figure 9, D and G), which has been associated with spatial memory (49). We also detected mCherry expression in the orbital frontal cortex (Supplemental Figure 9, E and G) and the amygdala but found that it was predominately limited to the medial divisions (Supplemental Figure 9, F and G). Several other brain regions also expressed low amounts of mCherry, but expression was inconsistent among mice (Supplemental Table 1). Therefore, the aBNST nociceptin–expressing neuron population that projects to the arcuate nucleus receives significant inputs from both homeostatic regions in the hypothalamus plus cortical and subcortical inputs that underlie a diverse array of behaviors that may be associated with feeding.

## Discussion

The control of feeding by AgRP/NPY neuron activity is sensitive to many homeostatic influences, and the nature of the hormonal and nutrient signals and hypothalamic neurocircuitry underlying such regulation has been well explored. It is increasingly recognized that sensory cues, reward signals, stress, learning, and memory may also affect AgRP/NPY neuron activity (7, 10, 11, 13, 14). However, little is known about the identity and function of the neuronal circuits that may be involved in this control. Here, we define a previously unrecognized aBNST neuronal pathway, outside of the classical homeostatic circuitry, that directly regulates AgRP/NPY activity, feeding, and body weight. In addition, these neurons receive inputs from several higher brain regions and may thus play a role in coordinating homeostatic and nonhomeostatic elements of feeding behavior.

We targeted the aBNST for several reasons: (a) it receives inputs from a diverse array of brain regions that may be involved in feeding (16, 20, 21); (b) it sends axonal projections to the arcuate nucleus, and retrograde mapping of AgRP neurons indicates a connection to the BNST (15, 16); and (c) lesioning studies, local pharmacological manipulation of the BNST, and optogenetic control of the aBNST alter body weight and feeding (18–20). Our finding that aBNST GABAergic neurons inhibit arcuate AgRP/NPY neurons and suppress food intake now implicates this circuit in the direct regulation of feeding. Although in vivo stimulation of aBNST nerve fibers could evoke transmitter release and inhibit other types of neurons in the immediate vicinity of the implanted optical fibers, the observed suppression of food intake is consistent with the phenotype seen with direct or synaptically induced AgRP/NPY neuronal inhibition (5, 7). aBNST GABAergic neurons have previously been implicated in feeding behavior, as optogenetic activation of their projections to LH glutamatergic neurons produced voracious feeding in satiated mice (18). However, subsequent studies have suggested that this circuitry may have a broader role in appetitive behaviors that is not necessarily specific to food (6, 50). In contrast, our studies identify an aBNST GABAergic pathway that specifically synapses with AgRP/NPY feeding neurons that may provide a direct input from higher brain centers to a key and well-established feeding circuit.

The aBNST is a complex region in terms of cytoarchitecture, synaptic connectivity, and the fast transmitters, neuropeptides, and receptors that are expressed within the various subnuclei (16, 20, 22, 47). Recent evidence has shown that expression of specific neuropeptides may underlie the functional specificity of different BNST GABAergic neuron populations involved in the regulation of emotional states (23). We were therefore interested to determine whether such a model may also operate with respect to the GABAergic aBNST population that regulated feeding via AgRP/NPY neurons. The opioid peptides dynorphin and nociceptin were attractive candidates, since they are highly expressed in the aBNST and have inhibitory effects on arcuate neuron populations (24, 26). Our finding that nociceptin- but not dynorphin-expressing neurons in the aBNST were synaptically connected to AgRP/NPY neurons suggests that a distinct neurochemically defined population may indeed regulate this arcuate nucleus cell population. Consistent with these observations, our RiboTag RNA-Seq studies demonstrated enrichment of nociceptin and depletion of dynorphin RNAs in these cells, further suggesting that they are a specific cell population. Our subsequent in vivo studies demonstrated that optogenetic stimulation of aBNST GABAergic nociceptin axons in the arcuate nucleus suppressed food intake, consistent with AgRP neuronal inhibition and with our data targeting the whole aBNST GABAergic cell population. In accordance with these findings, in vivo optogenetic activation of aBNST GABAergic nociceptin neurons led to suppression of AgRP neuron activity, suggesting a functional link between these events. Moreover, the ablation of aBNST GABAergic nociceptin neurons increased body weight and food intake, leading to increased adiposity. These changes, although not of the same magnitude, resemble the increases in body weight produced by chronic direct modulation of AgRP/NPY neurons, which alters feeding and energy expenditure (5). However, in our ablation studies, we did not directly assess AgRP neuron activity, and so the precise contribution of any disinhibition of AgRP neurons through ablation of aBNST nociceptin neurons remains to be defined.

The findings of an anorexigenic effect on activation of nociceptin-expressing neurons might seem at odds with the previous literature that describes an increase in feeding when pharmacological doses of nociceptin are delivered either intracerebroventricularly or into specific brain regions such as the arcuate nucleus (20, 28, 51). It has been suggested that this orexigenic effect occurs via inhibition of arcuate POMC and VMH neurons (26, 28, 29). However, injection of neuropeptides is unlikely to resemble the spatial and temporal pattern of physiological release but will instead likely induce long-lasting binding to nociceptin receptors throughout the arcuate nucleus, VMH, and other regions. Thus, the physiological outcome of pharmacological nociceptin administration is likely to be an amalgamated phenotype with perhaps the predominant effect occurring when the peptide acts upon a range of nociceptin target neurons, depending on the balance of activity at AgRP and POMC neurons. Indeed, simultaneous ablation of arcuate anorexigenic POMC and orexigenic AgRP/NPY neurons results in mild obesity, suggesting a dominance of POMC neurons in such manipulations (52).

Nociceptin is likely to have additional roles in feeding behavior, as its administration into the BNST prevents corticotropin-releasing hormone–induced stress and anorexia (51). In addition, nociceptin-expressing neurons in the central amygdala (CeA) promote the intake of highly palatable food but not normal chow, and nociceptin-expressing neurons in the ventral tegmental area (VTA) are also involved in reward behavior (53, 54). Intriguingly, CeA nociceptin–expressing neurons project to the ventral aBNST to alter reward and anxiety but not food intake. These findings contrast with our work on aBNST nociceptin–expressing neurons, which showed feeding and body weight phenotypes in mice on a chow diet. We also found no effects of the activation of these neurons upon anxiety-like behaviors, and our rabies tracing studies revealed limited connection with the CeA or the PVN that forms part of the hypothalamic-pituitary-adrenal axis underlying stress responses (55). Taken together, it is likely that the aBNST and CeA nociceptin circuits are distinct pathways that regulate different aspects of feeding behavior.

AgRP/NPY neuron activity drops prior to feeding, but the mechanisms underlying this observation and its physiological consequences are complex. The dynamic changes in AgRP/NPY neuron activity are thought to be primarily due to changes in the circulating levels of hormones and nutrients but are also influenced by sensory signals, indicating that anticipatory and perhaps other inputs are involved (7, 9–13). Several processes have been suggested to be mediated by the changes in AgRP/NPY activity, including the concept that this activity provides a negative teaching signal, suppresses appetitive behaviors, and provides a reward signal (7, 10, 13, 14). The rapid reductions in activity seen in homeostatic circuits may also be learned (13). In fiber photometric studies, we observed an increase in endogenous aBNST nociceptin neuron activity when mice began to eat but not when they approached a novel object. These data closely resemble the temporal changes in activity observed in GABAergic neurons in the DMH and in AgRP/NPY neurons, which increase and decrease their activity prior to feeding, respectively. Nevertheless, additional molecular studies such as those using loss-of-function approaches will be required to determine whether the activity of these presynaptic neurons underlies, in part, the reduction in AgRP activity during feeding. Indeed, there is a temporal discrepancy between altered endogenous nociceptin neuron activity (seconds to minutes) and the suppression of food intake (minutes to hours) induced by photostimulation, although such differences are likely to be due to our inability to detect small changes in food intake at earlier time points. In addition, the magnitude and time course of feeding suppression seen with optogenetic activation of DMH GABAergic neurons are more marked than what we observed with aBNST photostimulation (7). This may indicate that DMH neurons innervate and inhibit arcuate AgRP neurons to a greater extent than do aBNST neurons.

To determine whether synaptic inputs from other brain regions could provide the anatomical basis for this dynamic shift in activity, we undertook presynaptic tracing studies in aBNST GABAergic nociceptin neurons that projected to the arcuate nucleus. We used the AAV2(retro) serotype to express Cre recombinase specifically in aBNST GABAergic nociceptin neurons projecting to the mediobasal hypothalamus and then targeted these neurons with a glycoprotein-deficient rabies virus that expressed mCherry in the presynaptic neurons. As anticipated, given the high level of interconnectivity within the BNST, we observed substantial mCherry expression within both the aBNST and pBNST. We also observed substantial expression in the hypothalamus, with the POA being most connected. Interestingly, we detected very little expression in the arcuate nucleus despite the observation that AgRP/NPY neurons project to the aBNST to control feeding, suggesting that an alternate aBNST neuron population may mediate this effect (17). We also observed inputs from the subiculum and septum, which are major output regions of the hippocampus and could suggest that sensory or spatial memory is influencing the activity of these neurons (16). We also observed significant inputs from the cingulate cortex, which is implicated in a range of behaviors including those involving emotion, reward, and memory, as well as binge-eating disorders (56, 57). In contrast, we observed only scattered input from the medial and basomedial amygdala, no input from the basolateral amygdala, and very sparse input from the sensory cortical and hindbrain regions. This anatomical profile is therefore consistent with the targeting of the medial aBNST rather than the lateral aBNST, which would suggest that our approach was effectively capturing the amBNST region that projects to the arcuate nucleus (16, 22, 46). It is therefore possible that these additional inputs from higher brain regions may modulate the activity of aBNST nociceptin neurons as mice feed. In turn, this would regulate AgRP/NPY neuron activity, allowing these feeding neurons to be sensitive to a wide range of extrahypothalamic inputs. However, the functional significance of these connections has yet to be established and will require further study.

In summary, we have defined a previously unrecognized mechanism by which AgRP/NPY neuron activity, feeding, and body weight are modulated by a neuropeptide-defined GABAergic aBNST population. This circuitry is active during feeding and is connected to a range of hypothalamic and higher brain regions, and this may permit nonhomeostatic brain regions to direct the activity of a key homeostatic neuronal pathway. With a behavior that is as complex as feeding, which requires information from multiple neuronal circuits and homeostatic inputs, it is important to be able to process this information without losing the fidelity of the key inputs that initiate feeding. We therefore propose that aBNST GABAergic nociceptin neurons provide a gateway that integrates nonhomeostatic circuits into a single synaptic output regulating AgRP/NPY neuron activity and feeding.

## Methods

### Mice.

Vgat-Cre (*Slc32a1*tm2(Cre)Lowl/J), *Pdyn*-Cre (B6.129S-*Pdyn*tm1.1(Cre)Mjkr/LowlJ), Sf1-Cre (Tg(*Nr5a1*-Cre)7Lowl/J), *Agrp*-Cre (*Agrp*tm1(Cre)Lowl), *Npy*-hrGFP (B6.FVB-Tg(*Npy*-hrGFP)1Lowl/J), and *Pomc*-GFP (B6.Cg-Tg(*Pomc*-MAPT/Topaz)1RCK/J) mice were obtained from The Jackson Laboratory. BAC-transgenic mice (Tg(*Pnoc*-EGFP)#Uze) expressing EGFP under the transcriptional control of the *Pnoc* gene were generated using homologous recombination of the BAC and pronucleus injection of the modified BAC into fertilized oocytes. Eight clones of the BAC library RPCI-23 (Research Genetics) containing exon 2 of the *Pnoc* gene were obtained. RecA-mediated homologous recombination (58) was used to introduce EGFP into the *Pnoc* gene. The EGFP start codon was placed precisely into the position of the endogenous *Pnoc* start codon. To this end, 5′ and 3′ homology arms (0.7 and 1.5 kb in length) flanking the start codon and exon 2 of *Pnoc*, respectively, were generated by PCR. After 2 rounds of homologous recombination, BAC clones containing the desired recombination were identified by Southern blot analysis. Sizing of the modified BACs revealed that BAC 452H11 contained a sequence larger than 120 kb upstream of EGFP. This clone was chosen for pronucleus injection. Purified linearized BAC DNA was injected into the male pronuclei of fertilized friend virus B (FVB) oocytes. Two lines of mice were obtained and showed germline transmission. Details on the preparation of DNA and the injection procedure have been previously described (59).

Mice were bred on a C57Bl/6J background and maintained on a 12-hour light/12-hour dark cycle with free access to water and standard mouse chow (4.25% fat, RM3, Special Diet Services). Mice were housed in specific pathogen–free barrier facilities in individually ventilated cages of mixed genotypes. Male transgenic mice were age matched (3–5 months) and studied with their littermate controls. When possible, investigators were blinded to the genotype of the study animals. Mice were randomized by genotype to study groups, or a crossover design was used where indicated, and study cohorts were matched for initial body weight as appropriate. Mice were group housed (3–5 mice per cage) unless otherwise indicated.

### Genotyping.

Generic Cre recombinase (5′-AGCGATGGATTTCCGTCTCT-3′ and 5′-CACCAGCTTGCATGATCTCC-3′) and GFP (5′-AGCTAGCCACCATGGTGAGCAAGGGCGAGGAG-3′ and 5′-ATCTCGAGCTTGTACAGCTCGTCCATGCCG-3′) primers were used to genotype *Agrp*-Cre, Vgat-Cre, *Pdyn*-Cre, Sf1-Cre, *Pnoc*-EGFP, and *Pomc*-GFP mice. *Npy*-hrGFP mice were genotyped using the following primers: 5′-TATGTGGACGGGGCAGAAGATCCAGG-3′, 5′-CCCAGCTCACATATTTATCTAGAG-3′, and 5′-GGTGCGGTTGCCGTACTGGA-3′.

### Plasmids and viruses.

A Cre-dependent ChR2-mCherry plasmid [pAAV-EF1a-double-floxed-hChR2(H134R)-mCherry, Addgene plasmid 20297] and an flp-dependent ChR2-EYFP plasmid [pAAV-Ef1a-fDIO hChR2(H134R)-EYFP, Addgene plasmid 55639] were gifts from Karl Deisseroth (Howard Hughes Medical Institute, Stanford University, Stanford, California, USA) (60). hChR2(H134R)-mCherry was subcloned into the pAAV-Ef1a-fDIO hChR2(H134R)-EYFP vector using AscI and NheI restrictions sites to replace hChR2(H134R)-EYFP and make pAAV-Ef1a-fDIO hChR2(H134R)-mCherry. The sequence for jRGECO1a (pGP-CMV-NES-jRGECO1a; Addgene plasmid 61563) was a gift from Douglas Kim (Janelia Research Campus, Howard Hughes Medical Institute, Ashburn, Virginia, USA) and the GENIE project (41), and the sequence for the HA-tagged ribosomal protein (RiboTag) was provided by G. Stanley McKnight (University of Washington, Seattle, Washington, USA) (36, 37). Sequences were then synthesized by GeneArt (Life Technologies, Thermo Fisher Scientific) and subcloned into the pAAV-EF1a–double-floxed hChR2(H134R)-mCherry vector using AscI and NheI restriction sites to invert the sequence and replace ChR2-mCherry. An ires-mCherry sequence was also inserted upstream of the RiboTag sequence to make the resulting plasmids pAAV-EF1a–double-floxed jRGECO1a and pAAV-EF1a–double-floxed RiboTag-ires-mCherry. Plasmids for N-CretrcintG (pAAV-EF1a-N-CretrcintG, Addgene plasmid 69570), C-CreintG (pAAV-EF1a-C-CreintG, Addgene plasmid 69571), and DOG-flpo (pAAV-EF1a-Flp-DOG-NW, Addgene plasmid 75469) were gifts from Connie Cepko (Harvard Medical School, Boston, Massachusetts, USA) (34, 61). Cre-dependent caspase 3 (pAAV-flex-taCasp3-TEVp, Addgene plasmid 45580) was a gift from Nirao Shah (Stanford University, Stanford, California, USA) (42). Viral plasmids were commercially packaged at Vector Biolabs using the AAV1 or AAV2(retro) serotypes (44). Viral particles expressing Cre-dependent yellow fluorescent protein (YFP) (pAAV-EF1a-DIO EYFP, Addgene plasmid 27056-AV1) were a gift from Karl Deisseroth (Stanford University, Stanford, California, USA). AAV8-serotyped AAV particles expressing Cre-dependent GFP-tagged type-2 avian retroviral receptor (pAAV-EF1a-flex-GT, Addgene plasmid 26198) were a gift from Edward Callaway (The Salk Institute for Biological Sciences, La Jolla, California, USA). Nuclear-tagged N2c-glycoprotein (pAAV-hSyn-flex H2B-EGFP-P2A-N2cG, Addgene plasmid 126469) and synaptophysin-mCherry (pAAV-hSyn-flex synaptophysin mCherry) were serotyped with AAV1 and a glycoprotein-deficient rabies virus (CVS-N2c^ΔG^-mCherry, Addgene plasmid 126468) (43), pseudotyped with EnvA, and were gifts from Molly Strom and Troy Margrie (Sainsbury Wellcome Center, University College London, London, United Kingdom).

### Stereotactic surgery.

Age-matched (3–5 months of age) mice were placed in a Kopf stereotaxic frame, and AAV particles or rabies virus were injected bilaterally (or unilaterally where indicated) with a Hamilton syringe (0.3 μL/min, 0.15–0.3 μL per injection site) into either the aBNST (from bregma; anterior-posterior [AP], +0.30 mm; medial-lateral [ML], ± 0.65 mm, and dorsal-ventral [DV] from skull, –4.30 and –4.50 mm); the septum (AP, +0.30 mm; ML, ± 0.65 mm; DV, –3.00 and –3.20 mm); the arcuate nucleus (AP, –1.30 mm; ML, ± 0.40 mm; DV, –5.80 and –6.10 mm); or the VMH (AP, –1.35 mm; ML, ± 0.45 mm; DV, –5.70 and –6.00 mm). In optogenetic and photometric studies, ferrule-attached multimodal optical fibers (200-μm) were inserted bilaterally or unilaterally at a 10^o^ angle into (AP, –1.30 mm; ML, ± 1.31 mm; DV, –6.30 mm) or dorsal to (AP, –1.30 mm; ML, ± 1.15 mm; DV, –5.40 mm) the arcuate nucleus, or at a 5^o^ angle into the medial-dorsal region of the aBNST (AP, +0.30 mm; ML, ±1.15 mm; DV, –4.20 mm). Optical fiber tracts and viral expression were used to confirm correct stereotaxic placement in all mice postmortem.

### Optogenetics.

Cre-dependent ChR2-mCherry (1.7 × 10^13^ GC/mL) or YFP (1.2 × 10^13^ GC/mL) AAVs were injected into the aBNST. *Pnoc*-EGFP mice were also injected with AAV1 particles containing N-CretrcintG (1.8 × 10^13^ GC/mL) and C-CreintG (3.1 × 10^13^ GC/mL). Flp-dependent ChR2-mCherry (2.0 × 10^13^ GC/mL) and DOG-flpo (1.8 × 10^13^ GC/mL) AAVs were also unilaterally injected into the aBNST. Ferrule-attached optical fiber implants were coupled to a ferrule-attached optical fiber (200-μm diameter, multimodal) connected to a collimated rotary joint (Doric Lenses), as described previously (62). The rotary joint was connected via an optical fiber to a 473-nm laser (Vortran). To control the pattern of laser stimulation, an STG pulse generator (Multichannel Systems) was used to send 5-ms digital pulses (10 Hz for 3 seconds, repeated every 4 seconds) to the laser interlock function. Laser output at the end of the implanted ferrules ranged from 10 to 12 mW.

### Feeding behavior.

Four-month-old male Vgat-Cre, *Pnoc*-EGFP, and WT mice expressing Cre-dependent ChR2-mCherry or YFP were used to assess feeding. Mouse habituation commenced 2 weeks after stereotaxic surgery and consisted of daily handling, optical fiber tethering, and mock overnight fasting in the home cage. On the day of the test, overnight-fasted mice were tethered and placed in a clean home cage with free access to water. Laser stimulation began 10 minutes prior to the presentation of normal chow and continued for an additional 4 hours. Food consumption was assessed throughout the stimulation period and for a further 2 hours thereafter. Stimulated and nonstimulated mice were crossed over the following week.

### Anxiety behavior.

WT littermates and *Pnoc*-EGFP mice expressing Cre recombinase with and without ChR2-mCherry in the aBNST were used to assess anxiety-related behavior. Mouse habituation commenced 2 weeks after stereotaxic surgery and consisted of daily handling and optical fiber tethering. Mice were transferred to a novel room, tethered to a laser-attached optical fiber, and placed in a novel open-field arena (0.45 m open-top, square wooden box). A burst pattern (10 Hz for 3 seconds, repeated every 4 seconds) of laser stimulation was then immediately initiated, and mouse locomotion was video recorded and tracked using EthoVision (Noldus) software for 30 minutes. A week later, mice were tested on an Elevated Zero Maze (Panlab) during continuous (burst) laser stimulation for 10 minutes. Video tracking and manual scoring were used to determine the time spent and entries into the anxiogenic open zones.

### Fiber photometry.

Fiber photometry was performed on 4-month-old male *Pnoc*-EGFP mice injected with AAV particles containing N-CretrcintG (1.8 × 10^13^ GC/mL), C-CreintG (3.1 × 10^13^ GC/mL), and Cre-dependent jRGECO1a (0.9 × 10^13^ GC/mL) in the aBNST. In some studies, the arcuate nucleus of *Agrp*-Cre mice was injected with jRGECO1a (0.9 × 10^13^ GC/mL). Implanted optical fibers were coupled to a ferrule-attached optical fiber (200-μm diameter, multimodal) connected to a fiberoptometer (NPI Electronic). Briefly, light from a 565-nm light-emitting diode (LED) was passed through an excitation filter (545 ± 15 nm) and down the optical fiber via a dichroic mirror (transmission ≥570 nm). jRGECO1a fluorescence (peak ~600 nm) was transmitted back through the dichroic mirror, an emission filter (620 ± 30 nm), and into a photomultiplier. Voltage output was low-pass filtered at 340 Hz and sampled at 200 Hz using PClamp10 software. Continuous low-power (100–120 μW at the fiber tip) light was used to excite jRGECO1a. Any ambient and LED light that may have been captured by the photomultiplier was subtracted from fluorescence by generating templates acquired in between trials but recorded without attaching the optical fiber to the implanted ferrules. During photometry, mice were video recorded and tracked with EthoVision software, which was digitally coupled to the fiberoptometer to time-lock the behavior with fluorescence output.

Mouse habituation commenced 2 weeks after surgery and consisted of daily handling, optical fiber tethering, and exploration of an open-field arena (0.45 m open-top, square wooden box) with 2 circular pots (5-cm diameter) centered in the middle containing normal chow or a familiar object (chew block). A mock overnight fast was conducted in the home cage and in the open-field arena on consecutive weeks. One week later, overnight-fasted mice were tethered and placed in the arena containing 2 pots without food. After 10 minutes of arena habituation, the LED light was turned on and recordings commenced. After 10 minutes, a novel object (metal nut) was added to 1 pot, and then after another 10 minutes a food pellet (normal chow, ~1 cm^3^) was added to the remaining pot. Recordings were continued for an additional 10 minutes. Voltages were aligned to the time at which the mice approached the novel object or took the first bite of food. For studies investigating the effects of optogenetic activation of aBNST nociceptin neurons on AgRP neuron activity, after a 10-minute control period, the aBNST was photostimulated for 2 minutes during continuous excitation and recording of jRGECO1a activity in the arcuate nucleus. The change in fluorescence (ΔF) at each data point was divided by the median fluorescence (F) during baseline recordings (0.5–2 minutes before the behavioral event). The sample rate was then reduced to 1 Hz by IgorPro6 software for statistical purposes.

### Caspase-induced cell death.

Caspase-induced cell death was performed using 5-month-old male *Pnoc*-EGFP mice and WT littermate controls injected into the aBNST with AAV particles containing N-CretrcintG (1.1 × 10^13^ GC/mL), C-CreintG (1.9 × 10^13^ GC/mL), and Cre-dependent ChR2-mCherry (1.2 × 10^13^ GC/mL) with or without Cre-dependent caspase 3 (1.1 × 10^12^ GC/mL). Mice were group-housed and weighed weekly before and after stereotaxic surgery. Whole-body fat and lean mass were measured 6 weeks after surgery by EchoMRI analysis. Mice were also singly housed 4 weeks after surgery for at least 2 weeks before measurements of ad libitum food intake, as described previously (62). Postmortem PFA-fixed, 35-μm coronal slices containing the aBNST were used to count mCherry-positive somas in mice expressing ChR2-mCherry with or without caspase 3.

### RiboTag and RNA-Seq.

The RiboTag approach was performed as described previously (36, 37). AAV1 particles containing N-CretrcintG (1.8 × 10^13^ GC/mL), C-CreintG (3.1 × 10^13^ GC/mL), and Cre-dependent RiboTag-ires-mCherry (0.8 × 10^13^ GC/mL) were bilaterally injected into the aBNST of 4-month-old male *Pnoc*-EGFP mice. Four weeks after surgery, mice were culled, and the aBNST was dissected and homogenized in 800 μL polysome buffer (50 mM Tris, pH 7.5, 100 mM KCl, 12 mM MgCl_2_, 1% v/v IGEPAL, 1 mM DTT, 200 U/mL), RNasin (Promega), 1 mg/mL heparin, 100 μg/mL cycloheximide (Sigma-Aldrich), and a protease inhibitor cocktail (GE Healthcare). Homogenates were centrifuged at 10,000 ×*g* for 10 minutes at 4^o^C to create a postmitochondrial supernatant. Supernatant (40 μL) was set aside to extract total aBNST RNA, and 750 μL of the remaining supernatant was incubated overnight at 4^o^C with anti-HA–coupled protein G beads. Magnetically captured beads were washed in high-salt buffer (50 mM Tris, pH 7.5, 300 mM KCl, 12 mM MgCl_2_, 1% v/v IGEPAL, 1 mM DTT, 100 μg/mL cycloheximide) and polysomes eluted with lysis buffer (QIAGEN, catalog 74106). RNA was extracted from the eluted samples and homogenate supernatant using an RNeasy Mini Kit (QIAGEN, catalog 74106). RNA was treated with DNAase (QIAGEN, catalog 79254). Total RNA (20 ng) was depleted of rRNA using a NEBNext rRNA Depletion Kit (New England BioLabs [NEB], E6310), RNA libraries were prepared using the NEBNext Ultra II Directional RNA Library Prep Kit for Illumina (NEB, E7760), and PCR enrichment was performed with NEBNext Multiplex Oligos for Illumina, set 1 (NEB, E7335). Paired-end, 100-bp sequences were obtained using an Illumina HiSeq 2500 (v4 reagents) Kit. Raw RNA-Seq reads were mapped with STAR aligner (version 020201) against the Ensembl mouse genome reference sequence assembly (mm10) and gencode vM19 gene annotations (63). Differential expression analysis was performed on the counts data using the Bioconductor DESeq2 package (version 1.22.2). The DESeq2 package uses negative binomial model-to-model read counts and then performs statistical tests for differential expression of genes (64). Raw *P* values were adjusted for multiple testing using the Benjamini-Hochberg procedure. Data were deposited in the NCBI’s GEO database (GEO GSE135982).

### Ex vivo electrophysiology and imaging.

AAV particles containing Cre-dependent ChR2-mCherry (1.7 × 10^13^ GC/mL) or jRGECO1a (0.9 × 10^13^ GC/mL) with or without N-CretrcintG (1.8 × 10^13^ GC/mL) and C-CreintG (3.1 × 10^13^ GC/mL) were injected into transgenic mice expressing Vgat-Cre, *Pdyn*-Cre, Sf1-Cre, or *Pnoc*-EGFP that were crossed with either *Pomc*-GFP or *Npy*-hrGFP mice. Ex vivo slices were maintained at room temperature (22°C–25°C) in an external solution containing 125 mM NaCl, 2.5 mM KCl, 1.25 mM NaH_2_PO_4_, 25 mM NaHCO_3_, 2 mM CaCl_2_, 1 mM MgCl_2_, 10 mM d-glucose, and 15 mM d-mannitol, equilibrated with 95% O_2_, 5% CO_2_ (pH 7.4). Whole-cell current-clamp recordings were made using borosilicate glass pipettes (4–8 MΩ) containing 130 mM potassium gluconate, 10 mM KCl, 0.5 mM EGTA, 1 mM NaCl, 0.28 mM CaCl_2_, 3 mM MgCl_2_, 3 mM Na_2_ATP, 0.3 mM GTP, 14 mM phosphocreatine, and 10 mM HEPES (pH 7.2). Nociceptin (Tocris) was bath applied and input resistance monitored by periodic hyperpolarizing current pulses. Synaptic release from ChR2-expressing axonal terminals was evoked by pulses (4-ms) of light (488 nm) generated by a monochromator (Polychrome V, TILL). A burst-stimulating protocol (10 Hz for 3 seconds, repeated every 4 seconds) was used to assess changes in spontaneous action potential firing frequency. jRGECO1a fluorescence was excited by 50-ms pulses of 590 nm light at 0.5 Hz using a monochromator. Depolarizing current injections were used to evoke action potential firing while simultaneously recording fluorescence intensity.

Whole-cell voltage-clamp recordings were made at –70 mV in a modified internal solution with potassium gluconate replaced with CsCl (130 mM), in which excitatory and inhibitory synaptic currents are observed as inward currents. Synaptic currents were photostimulated (4-ms pulse of 488 nm light) at 0.5 Hz. Bath applications of 20 μM (+)-bicuculline or 5 μM 2,3-dihydroxy-6-nitro-7-sulfamoyl-benzo(f)quinoxaline-2,3-dione (NBQX) with 50 μM d-2-amino-5-phosphonopentanoate (d-AP5) were used to distinguish GABAergic or glutamatergic events, respectively.

### Imaging, IHC, and ISH.

Mice were perfused with paraformaldehyde (4% w/v), and frozen coronal sections (30- to 35-μm-thick) were cut as previously described (65). aBNST sections were incubated with rabbit anti-nociceptin (1:500; Abcam, catalog ab10277), and detection was performed using a secondary antibody coupled to Alexa Fluor 594 (1:200, Invitrogen, Thermo Fisher Scientific, catalog A21442). Fluorescence images were captured using an Olympus IX70 microscope. ISH on mouse brain slices was performed using digoxigenin-labeled *Pnoc* antisense mRNA (mRNA 1-932; NCBI reference sequence NM_010932).

### Statistics.

Statistical significance was calculated using a repeated-measures 2-way ANOVA followed by Sidak’s post hoc analysis or a Student’s 2-tailed paired, unpaired, or 1-sample *t* test. Data are expressed as the mean ± SEM. A *P* value of less than 0.05 was considered significant.

### Study approval.

All animal studies were performed in accordance with the United Kingdom Animals (Scientific Procedures) Act (1986) and amended regulations (2012) and were approved by Imperial College Animal Welfare and Ethical Review Body. The experiments and findings described in this study were designed and reported following the Animal Research: Reporting of In Vivo Experiments (ARRIVE) guidelines for animal experiment reporting (66).

## Author contributions

MAS, AIC, JAG, PV, EEI, and PC performed experiments. HUZ contributed reagents. MAS and SK analyzed data. MAS and DJW developed the concepts, supervised the work, and wrote the manuscript. DJW secured funding. All authors contributed to the editing of the manuscript.

## Supplementary Material

Supplemental data

## Version 1 09/26/2019

In-Press Preview

## Version 2 11/18/2019

Electronic publication

## Version 3 01/02/2020

Print issue publication

## References

[1] Andermann ML, Lowell BB (2017). Toward a wiring diagram understanding of appetite control. Neuron.

[2] Denis RG (2015). Palatability can drive feeding independent of AgRP neurons. Cell Metab.

[3] Sternson SM, Nicholas Betley J, Cao ZF (2013). Neural circuits and motivational processes for hunger. Curr Opin Neurobiol.

[4] Aponte Y, Atasoy D, Sternson SM (2011). AGRP neurons are sufficient to orchestrate feeding behavior rapidly and without training. Nat Neurosci.

[5] Krashes MJ (2011). Rapid, reversible activation of AgRP neurons drives feeding behavior in mice. J Clin Invest.

[6] Burnett CJ (2016). Hunger-driven motivational state competition. Neuron.

[7] Garfield AS (2016). Dynamic GABAergic afferent modulation of AgRP neurons. Nat Neurosci.

[8] Krashes MJ (2014). An excitatory paraventricular nucleus to AgRP neuron circuit that drives hunger. Nature.

[9] Betley JN (2015). Neurons for hunger and thirst transmit a negative-valence teaching signal. Nature.

[10] Beutler LR, Chen Y, Ahn JS, Lin YC, Essner RA, Knight ZA (2017). Dynamics of gut-brain communication underlying hunger. Neuron.

[11] Chen Y, Lin YC, Kuo TW, Knight ZA (2015). Sensory detection of food rapidly modulates arcuate feeding circuits. Cell.

[12] Mandelblat-Cerf Y (2015). Arcuate hypothalamic AgRP and putative POMC neurons show opposite changes in spiking across multiple timescales. Elife.

[13] Su Z, Alhadeff AL, Betley JN (2017). Nutritive, post-ingestive signals are the primary regulators of AgRP neuron activity. Cell Rep.

[14] Chen Y, Lin YC, Zimmerman CA, Essner RA, Knight ZA (2016). Hunger neurons drive feeding through a sustained, positive reinforcement signal. Elife.

[15] Wang D (2015). Whole-brain mapping of the direct inputs and axonal projections of POMC and AgRP neurons. Front Neuroanat.

[16] Dong HW, Swanson LW (2006). Projections from bed nuclei of the stria terminalis, anteromedial area: cerebral hemisphere integration of neuroendocrine, autonomic, and behavioral aspects of energy balance. J Comp Neurol.

[17] Betley JN, Cao ZF, Ritola KD, Sternson SM (2013). Parallel, redundant circuit organization for homeostatic control of feeding behavior. Cell.

[18] Jennings JH, Rizzi G, Stamatakis AM, Ung RL, Stuber GD (2013). The inhibitory circuit architecture of the lateral hypothalamus orchestrates feeding. Science.

[19] Rollins BL, Stines SG, King BM (2006). Role of the stria terminalis in food intake and body weight in rats. Physiol Behav.

[20] Kash TL (2015). Neuropeptide regulation of signaling and behavior in the BNST. Mol Cells.

[21] Ch’ng S, Fu J, Brown RM, McDougall SJ, Lawrence AJ (2018). The intersection of stress and reward: BNST modulation of aversive and appetitive states. Prog Neuropsychopharmacol Biol Psychiatry.

[22] Gungor NZ, Paré D (2016). Functional heterogeneity in the bed nucleus of the stria terminalis. J Neurosci.

[23] Giardino WJ, Eban-Rothschild A, Christoffel DJ, Li SB, Malenka RC, de Lecea L (2018). Parallel circuits from the bed nuclei of stria terminalis to the lateral hypothalamus drive opposing emotional states. Nat Neurosci.

[24] Zhang X, van den Pol AN (2013). Direct inhibition of arcuate proopiomelanocortin neurons: a potential mechanism for the orexigenic actions of dynorphin. J Physiol (Lond).

[25] Bewick GA (2005). Hypothalamic cocaine- and amphetamine-regulated transcript (CART) and agouti-related protein (AgRP) neurons coexpress the NOP1 receptor and nociceptin alters CART and AgRP release. Endocrinology.

[26] Wagner EJ, Rønnekleiv OK, Grandy DK, Kelly MJ (1998). The peptide orphanin FQ inhibits beta-endorphin neurons and neurosecretory cells in the hypothalamic arcuate nucleus by activating an inwardly-rectifying K+ conductance. Neuroendocrinology.

[27] Li C (2012). Presynaptic inhibition of gamma-aminobutyric acid release in the bed nucleus of the stria terminalis by kappa opioid receptor signaling. Biol Psychiatry.

[28] Hernandez J, Fabelo C, Perez L, Moore C, Chang R, Wagner EJ (2019). Nociceptin/orphanin FQ modulates energy homeostasis through inhibition of neurotransmission at VMN SF-1/ARC POMC synapses in a sex- and diet-dependent manner. Biol Sex Differ.

[29] Chee MJ, Price CJ, Statnick MA, Colmers WF (2011). Nociceptin/orphanin FQ suppresses the excitability of neurons in the ventromedial nucleus of the hypothalamus. J Physiol (Lond).

[30] Lein ES (2007). Genome-wide atlas of gene expression in the adult mouse brain. Nature.

[31] Boom A (1999). Distribution of the nociceptin and nocistatin precursor transcript in the mouse central nervous system. Neuroscience.

[32] Ikeda K, Watanabe M, Ichikawa T, Kobayashi T, Yano R, Kumanishi T (1998). Distribution of prepro-nociceptin/orphanin FQ mRNA and its receptor mRNA in developing and adult mouse central nervous systems. J Comp Neurol.

[33] Neal CR, Mansour A, Reinscheid R, Nothacker HP, Civelli O, Watson SJ (1999). Localization of orphanin FQ (nociceptin) peptide and messenger RNA in the central nervous system of the rat. J Comp Neurol.

[34] Tang JC (2015). Cell type-specific manipulation with GFP-dependent Cre recombinase. Nat Neurosci.

[35] Tang JC (2013). A nanobody-based system using fluorescent proteins as scaffolds for cell-specific gene manipulation. Cell.

[36] Sanz E, Yang L, Su T, Morris DR, McKnight GS, Amieux PS (2009). Cell-type-specific isolation of ribosome-associated mRNA from complex tissues. Proc Natl Acad Sci U S A.

[37] Sanz E, Quintana A, Deem JD, Steiner RA, Palmiter RD, McKnight GS (2015). Fertility-regulating Kiss1 neurons arise from hypothalamic POMC-expressing progenitors. J Neurosci.

[38] Sweeney P, Li C, Yang Y (2017). Appetite suppressive role of medial septal glutamatergic neurons. Proc Natl Acad Sci USA.

[39] Sweeney P, Yang Y (2015). An excitatory ventral hippocampus to lateral septum circuit that suppresses feeding. Nat Commun.

[40] Sweeney P, Yang Y (2016). An inhibitory septum to lateral hypothalamus circuit that suppresses feeding. J Neurosci.

[41] Dana H (2016). Sensitive red protein calcium indicators for imaging neural activity. Elife.

[42] Yang CF (2013). Sexually dimorphic neurons in the ventromedial hypothalamus govern mating in both sexes and aggression in males. Cell.

[43] Reardon TR (2016). Rabies virus CVS-N2c(ΔG) strain enhances retrograde synaptic transfer and neuronal viability. Neuron.

[44] Tervo DG (2016). A designer AAV variant permits efficient retrograde access to projection neurons. Neuron.

[45] Avery SN, Clauss JA, Winder DG, Woodward N, Heckers S, Blackford JU (2014). BNST neurocircuitry in humans. Neuroimage.

[46] Dong HW, Swanson LW (2004). Organization of axonal projections from the anterolateral area of the bed nuclei of the stria terminalis. J Comp Neurol.

[47] Lebow MA, Chen A (2016). Overshadowed by the amygdala: the bed nucleus of the stria terminalis emerges as key to psychiatric disorders. Mol Psychiatry.

[48] McDonald AJ (1998). Cortical pathways to the mammalian amygdala. Prog Neurobiol.

[49] Vann SD, Aggleton JP, Maguire EA (2009). What does the retrosplenial cortex do?. Nat Rev Neurosci.

[50] Navarro M (2016). Lateral hypothalamus GABAergic neurons modulate consummatory behaviors regardless of the caloric content or biological relevance of the consumed stimuli. Neuropsychopharmacology.

[51] Ciccocioppo R, Biondini M, Antonelli L, Wichmann J, Jenck F, Massi M (2002). Reversal of stress- and CRF-induced anorexia in rats by the synthetic nociceptin/orphanin FQ receptor agonist, Ro 64-6198. Psychopharmacology (Berl).

[52] Xu AW (2005). Effects of hypothalamic neurodegeneration on energy balance. PLoS Biol.

[53] Hardaway JA (2019). Central amygdala prepronociceptin-expressing neurons mediate palatable food consumption and reward. Neuron.

[54] Parker KE (2019). A paranigral VTA nociceptin circuit that constrains motivation for reward. Cell.

[55] Stephens MA, Wand G (2012). Stress and the HPA axis: role of glucocorticoids in alcohol dependence. Alcohol Res.

[56] Stevens FL, Hurley RA, Taber KH (2011). Anterior cingulate cortex: unique role in cognition and emotion. J Neuropsychiatry Clin Neurosci.

[57] Geliebter A, Benson L, Pantazatos SP, Hirsch J, Carnell S (2016). Greater anterior cingulate activation and connectivity in response to visual and auditory high-calorie food cues in binge eating: Preliminary findings. Appetite.

[58] Yang XW, Model P, Heintz N (1997). Homologous recombination based modification in Escherichia coli and germline transmission in transgenic mice of a bacterial artificial chromosome. Nat Biotechnol.

[59] Zeilhofer HU (2005). Glycinergic neurons expressing enhanced green fluorescent protein in bacterial artificial chromosome transgenic mice. J Comp Neurol.

[60] Fenno LE (2014). Targeting cells with single vectors using multiple-feature Boolean logic. Nat Methods.

[61] Tang JC (2016). Detection and manipulation of live antigen-expressing cells using conditionally stable nanobodies. Elife.

[62] Viskaitis P (2017). Modulation of SF1 neuron activity coordinately regulates both feeding behavior and associated emotional states. Cell Rep.

[63] Dobin A (2013). STAR: ultrafast universal RNA-seq aligner. Bioinformatics.

[64] Love MI, Huber W, Anders S (2014). Moderated estimation of fold change and dispersion for RNA-seq data with DESeq2. Genome Biol.

[65] Choudhury AI (2005). The role of insulin receptor substrate 2 in hypothalamic and beta cell function. J Clin Invest.

[66] Kilkenny C, Browne WJ, Cuthill IC, Emerson M, Altman DG (2010). Improving bioscience research reporting: the ARRIVE guidelines for reporting animal research. PLoS Biol.

