# Supplementary material for: Extrahypothalamic GABAergic nociceptin–expressing neurons regulate AgRP neuron activity to control feeding behavior
Source: J Clin Invest. 2019 Nov 18;130(1):126–42. doi: 10.1172/JCI130340 (PMC6934207; doi:10.1172/JCI130340)
Supplement: Supplemental data [file jci-130-130340-s045.pdf]

Supplemental Data

Supplemental Table 1.

Brain Regions	Expression level (%)
Cortex	
Prelimbic area	0.1 ± 0.1
Infralimbic area	0.1 ± 0.0
Entorhinal cortex	0.1 ± 0.1
Piriform cortex	0.1 ± 0.1
Somatosensory cortex	0.1 ± 0.1
Amygdala	
Posterior cortical amygdala nucleus	0.2 ± 0.1
Anterior amygdaloid area	0.1 ± 0.1
Amygdalohippocampus	0.1 ± 0.0
Hippocampus	
CA1	0.5 ± 0.3
Pyramidal cell layer of hippocampus	0.5 ± 0.4
'Other' forebrain regions	
Zona inserta	1.2 ± 1.2
Sub-inserta nucleus	0.8 ± 0.8
Nucleus horizontal vertical diagonal band	0.6 ± 0.3
Medial habinula	0.4 ± 0.4
Mediodorsal thalamic nucleus	0.3 ± 0.3
Midbrain/brainstem	
Substantia nigra	0.9 ± 0.6
Periaquaductal grey area	0.7 ± 0.5
Dorsal Raphe	0.3 ± 0.1
Reticulotegmental nucleus of pons	0.3 ± 0.3
Posterodorsal tegmental nucleus	0.3 ± 0.3
Superior colliculius	0.3 ± 0.3
Central grey of pons	0.2 ± 0.2
Ventral tegmental area	0.1 ± 0.1
Premedian raphe nucleus	0.1 ± 0.1
Intermediate layer superior colliculus	0.1 ± 0.1
Pontine recticular nucleus	0.1 ± 0.1
Interpeduncular nucleus	0.1 ± 0.0

Supplemental Table 1. Monosynaptic rabies tracing of aBNST nociceptin pre-synaptic neurons. Expression is presented as the percentage number of mCherry somas identified outside the BNST. Mean ± SEM from 3 mice.

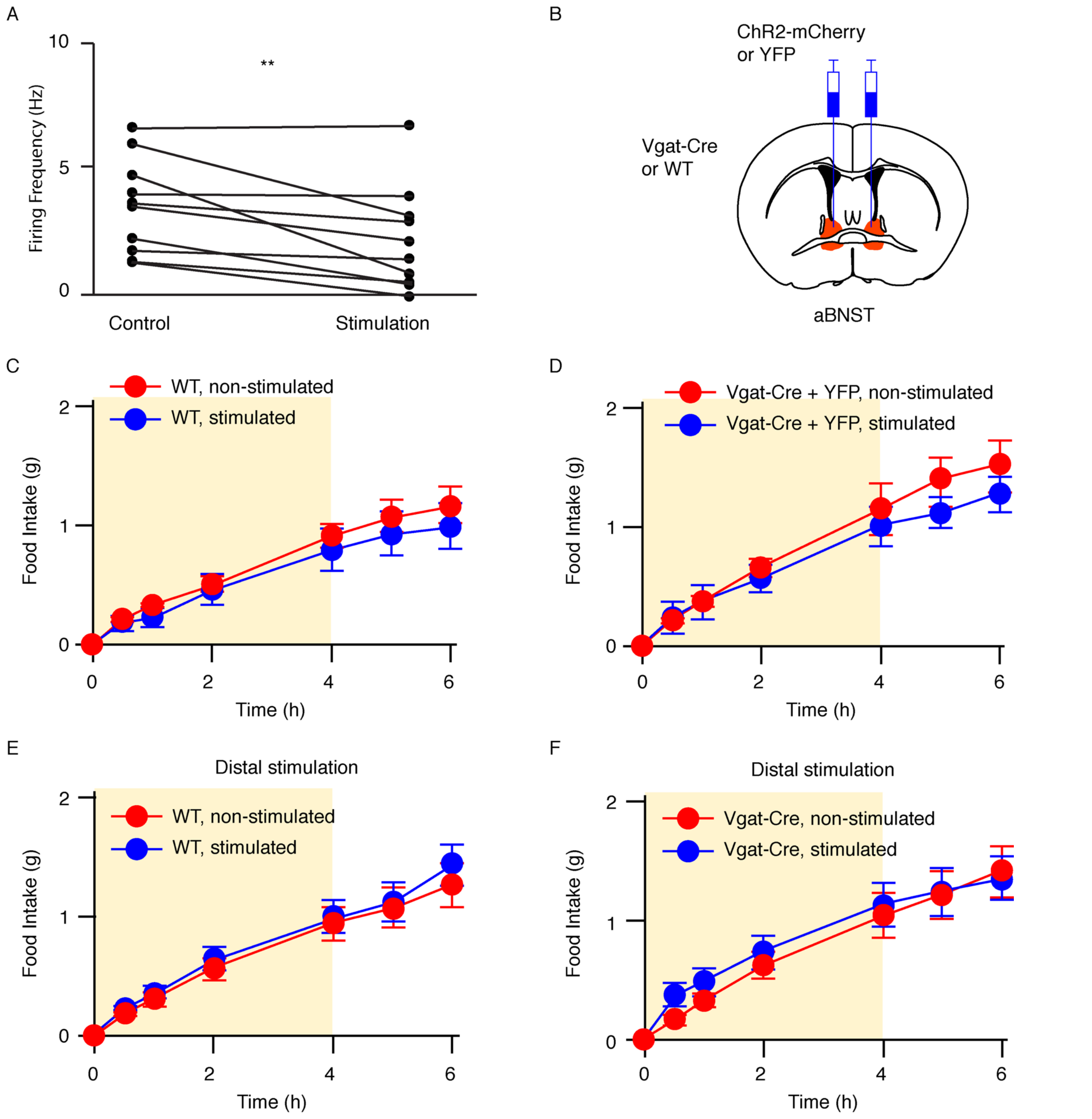

Supplemental Figure 1. Optogenetic stimulation does not suppress feeding in wild-type Vgat-Cre littermate mice.
(**A**) Graph showing the pairing of action potential frequency in arcuate NPY neurons (n = 10 neurons from 3 mice) before (control) and during photostimulation of aBNST Vgat axons. ** p<0.01 (paired t-test; t (9) = 3.33, p = 0.009). (**B**) Diagram of the aBNST in Vgat-Cre or wild-type (WT) mice injected with either cre-dependent ChR2-mCherry or YFP. (**C-F**) Cumulative food intake following an overnight fast during (shaded area) and after photostimulation in WT (**C** and **E**) or Vgat-Cre (**D** and **F**) mice injected with ChR2-mCherry (**C**,**E** and **F**) or YFP (**D**) into the aBNST. Food intake was measured without (non-stimulated, red) and with photostimulation (stimulated, blue) by optical fibers placed within (**C** and **D**) or ~0.9 mm dorsal (**E** and **F**) to the arcuate nucleus. Mean ± SEM. (**C**) N = 9 mice, 2-way RM ANOVA (Interaction: f (6,96) = 0.18, p = 0.98; stimulation: f (1,16) = 0.14, p = 0.71). (**D**) N= 4 mice, 2-way RM ANOVA (Interaction: f (6,36) = 0.74, p = 0.62; stimulation: f (1,6) = 0.47, p = 0.52). (**E**) N = 10 mice, 2-way RM ANOVA (Interaction: f (6,108) = 0.23, p = 0.97; stimulation: f (1,18) = 0.32, p = 0.58). (**F**) N = 9 mice, 2-way RM ANOVA (Interaction: f (6,96) = 0.58, p = 0.75; stimulation: f (1,16) = 0.22, p = 0.64).

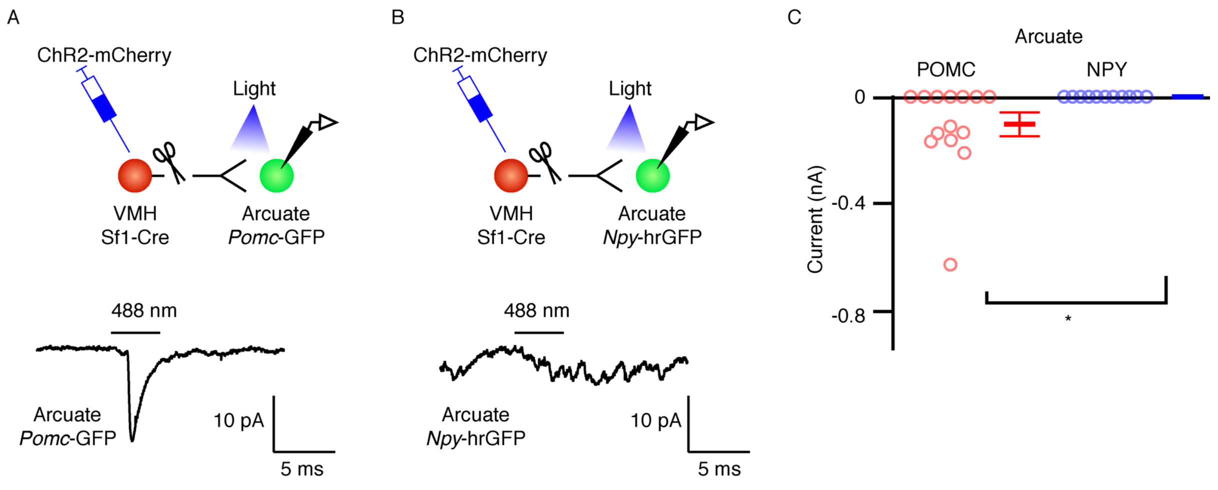

Supplemental Figure 2. VMH Sf1 neurons do not synapse with arcuate NPY neurons. (**A** and **B**) Diagrammatic representation of Sf1-Cre mice crossed with *Pomc*-GFP (**A**, top) or *Npy*-hrGFP (**B**, top) with the VMH injected with ChR2-mCherry AAV. Photostimulated currents are shown in arcuate POMC neurons (**A**, bottom, n = 14) but not NPY neurons (**B**, bottom, n = 11). (**C**) Evoked synaptic currents in arcuate POMC (n = 14 neurons from 4 mice) and NPY (n = 11 neurons from 4 mice) neurons. Mean ± SEM, * $p<0.05$ (unpaired t-test; $t(23) = 2.15$, $p = 0.046$).

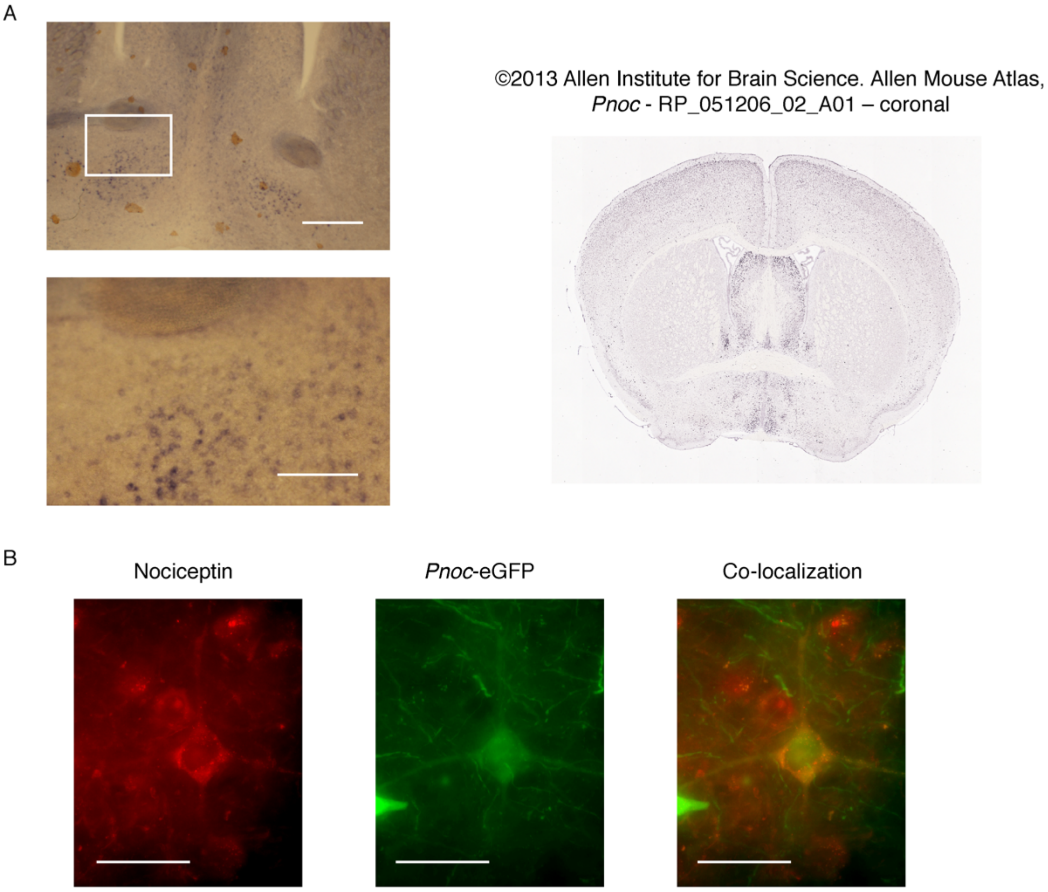

Supplemental Figure 3. aBNST expression of *Pnoc*.
(**A**) In situ hybridization of *Pnoc* in the aBNST, septum and preoptic area (left, n = 3) compared with *Pnoc* expression published in the Allen Mouse Brain Atlas (right, ©2013 Allen Institute for Brain Science; http://mouse.brain-map.org/experiment/show/75038402; *Pnoc*-RP_051206_02_A01–coronal). Scale bars are 500 μm and 200 μm for low and high magnification images, respectively. (**B**) Representative immunohistochemical images of the aBNST for nociceptin (red, left), eGFP driven by the *Pnoc* promoter (green, middle) and co-localization (right), n = 3. Scale bars are 20 μm.

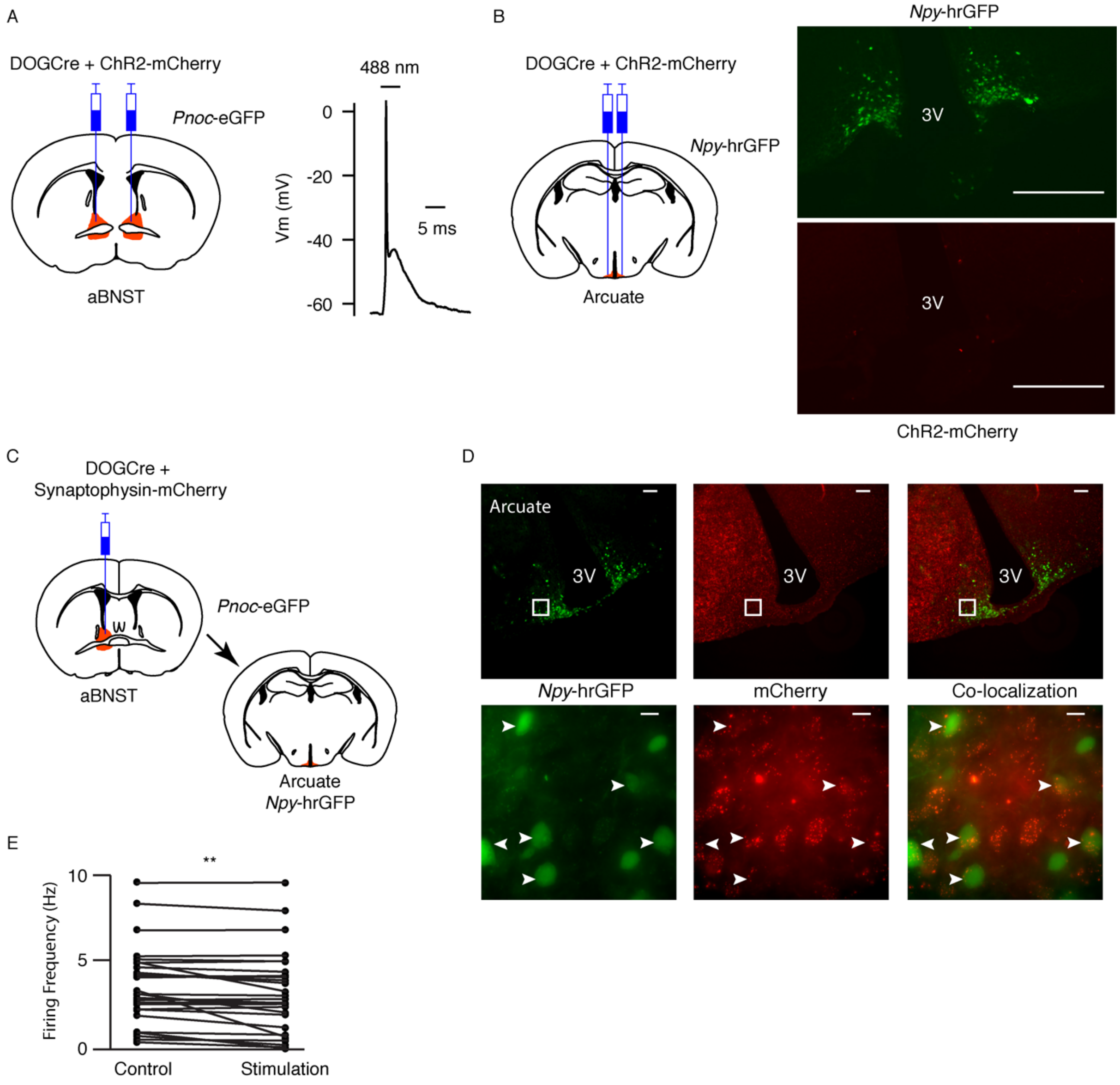

Supplemental Figure 4. aBNST *Pnoc* axons co-localize with arcuate NPY neurons.
(**A**) Diagram of the aBNST in *Pnoc*-eGFP mice injected with DOGCre and ChR2-mCherry (left) and voltage trace (right) from a photostimulated ChR2-mCherry expressing aBNST neuron (n = 3). (**B**) Diagram (left) of an arcuate neuronal section injected with DOGCre and ChR2-mCherry AAVs into *Npy*-hrGFP mice. Image of hrGFP expression (green, right-top) which does not lead to cre-dependent mCherry expression (red, right-bottom) in the arcuate nucleus, n = 6. Scale bars are 200 μm. (**C**) Line diagram of the aBNST and arcuate nucleus in *Pnoc*-eGFP mice crossed with *Npy*-hrGFP mice. The aBNST was unilaterally injected with AAVs containing DOG-Cre and cre-dependent synaptophysin fused to mCherry. (**D**) Expression of *Npy*-hrGFP (left), mCherry (middle) and co-localization (right) in the arcuate nucleus at low (top) and high (bottom) magnification corresponding to the boxed regions (n = 3). Arrows represent co-localization between *Npy*-hrGFP and synaptophysin-mCherry. Scale bars are 100 μm for low and 10 μm for high magnification images. 3V, 3rd ventricle. (**E**) Graph showing the pairing of action potential frequency in arcuate NPY neurons (n = 28 neurons from 9 mice) before (control) and during photo-stimulation of aBNST *Pnoc* axons. ** $p<0.01$ (paired t-test; $t(27) = 3.33$, $p = 0.003$).

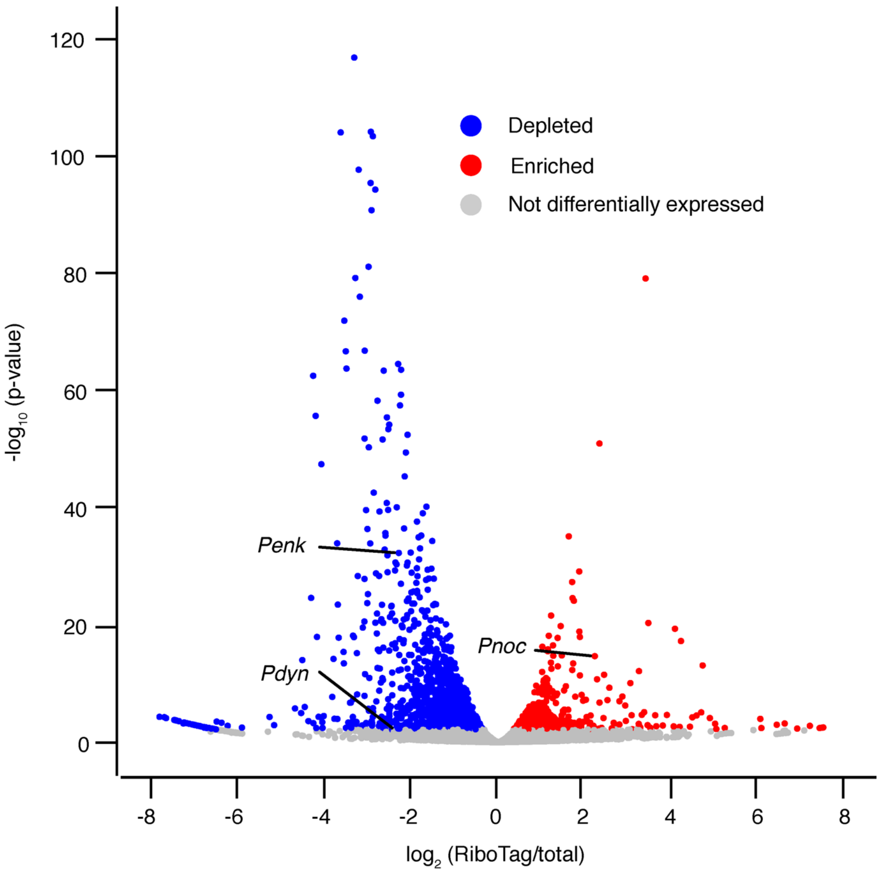

Supplemental Figure 5. RiboTag enrichment of RNA in *Pnoc* aBNST neurons.
Volcano plot of the fold-change between RiboTagged *Pnoc*-eGFP neurons versus total extracted RNA in the aBNST against statistical significance (Binomial Wald test, $p<0.05$ corrected for multiple testing by the Benjamin-Hochberg algorithm). Genes depleted are shown in blue, enriched in red and not differentially expressed in gray (n = 2 pools from 6 mice).

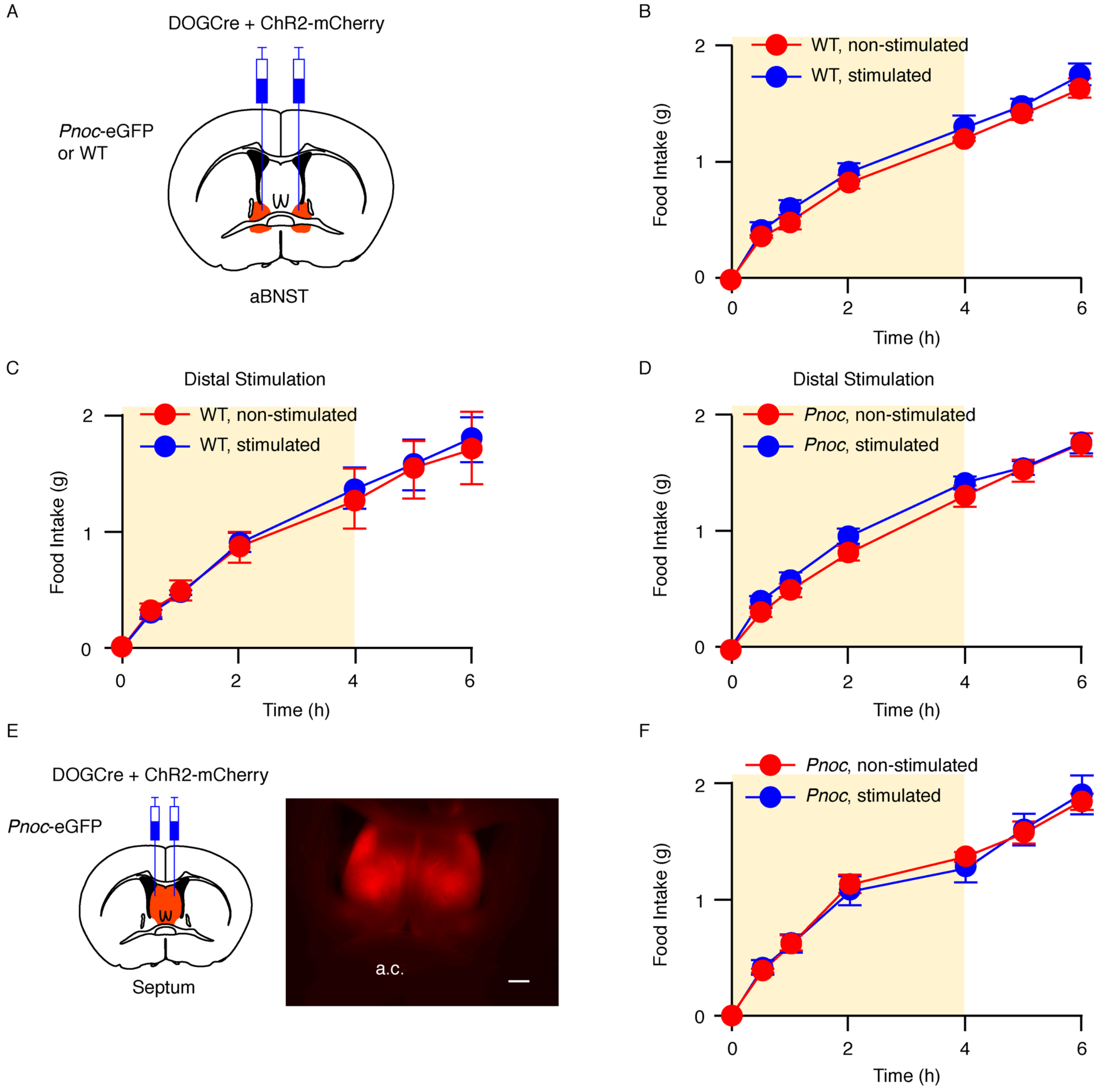

Supplemental Figure 6. Optogenetic stimulation does not suppress feeding in wild-type *Pnoc*-eGFP littermate mice.
(**A**) Line diagram of the aBNST in *Pnoc*-eGFP or wild-type (WT) mice injected with DOGCre and ChR2-mCherry. (**B-D**) Cumulative food intake following an overnight fast in WT (**B** and **C**) and *Pnoc*-eGFP (**D**) littermate mice injected with DOGCre and ChR2-mCherry into the aBNST during (shaded area) and after photostimulation of the arcuate nucleus. Food intake was measured without (non-stimulated, red) and with photostimulation (stimulated, blue) by optical fibers placed within (**B**) or ~0.6 mm dorsal (**C** and **D**) to the arcuate nucleus. Mean ± SEM. (**B**) N = 4 mice. 2-way RM ANOVA (Interaction: f (6,36) = 0.51, p = 0.80; stimulation: f (1,6) = 1.64, p = 0.25). (**C**) N = 4 mice. 2-way RM ANOVA (Interaction: f (6,36) = 0.09, p = 1.00; stimulation: f (1,6) = 0.03, p = 0.87). (**D**) N = 7 mice. 2-way RM ANOVA (Interaction: f (6,72) = 0.60, p = 0.73; stimulation: f (1,12) = 1.06, p = 0.32). (**E**) Line diagram of the septum (shaded red) in *Pnoc*-eGFP mice (left) injected with DOGCre and ChR2-mCherry into the septum. Representative expression of ChR2-mCherry is shown in the right panel (n = 5). Scale bar is 200 μm. a.c., anterior commissure. (**F**) Cumulative food intake in *Pnoc*-eGFP mice injected with ChR2-mCherry into the septum during (shaded area) and after photostimulation of the arcuate nucleus. Food intake was measured without (non-stimulated, red) and with photostimulation (stimulated, blue). Mean ± SEM, n = 5 mice. 2-way RM ANOVA (Interaction: f (6,48) = 0.30, p = 0.93; stimulation: f (1,8) = 0.02, p = 0.88).

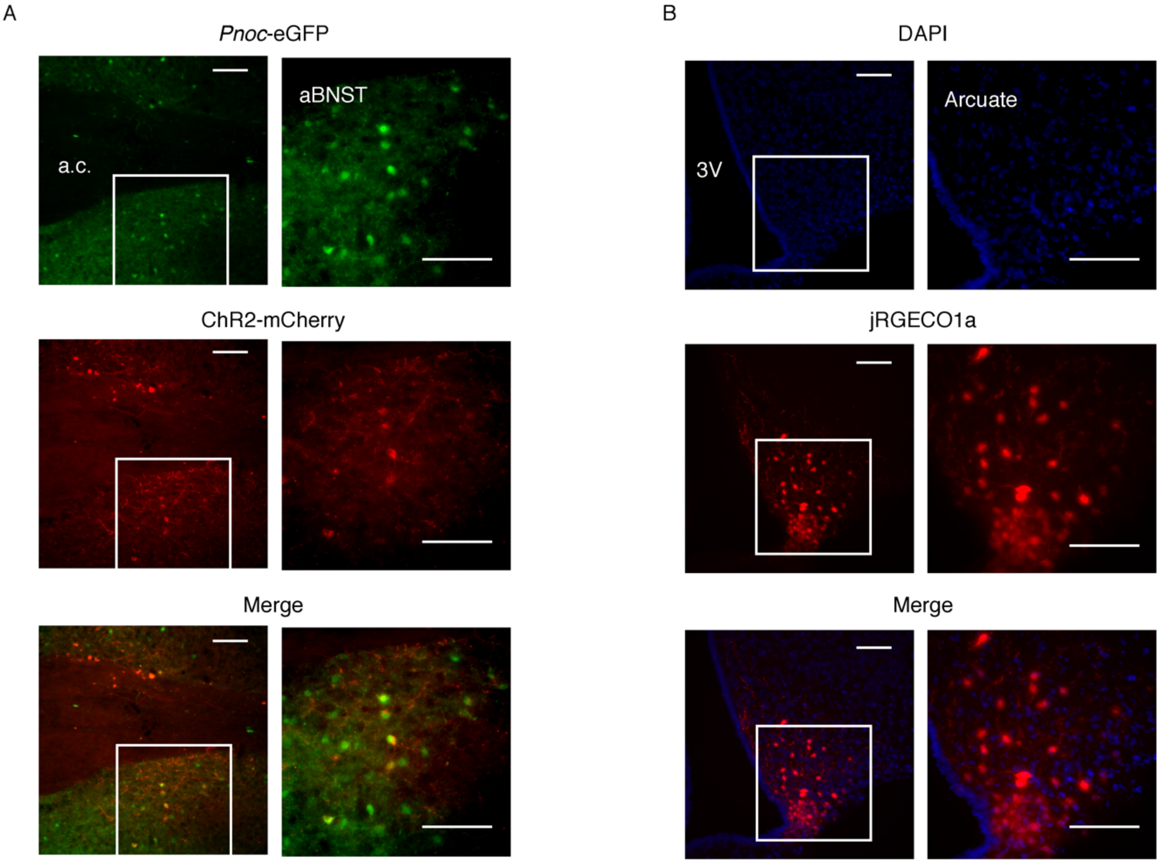

Supplemental Figure 7. Expression of ChR2 in aBNST and jRGECO1a in the arcuate nucleus. (**A** and **B**) Representative florescent images of the aBNST (**A**) and arcuate nucleus (**B**) in *Pnoc*-eGFP mice inter-crossed with *Agrp*-Cre mice (n = 5). The aBNST was injected with DOG-flpo and flp-dependent ChR2-mCherry and the arcuate with cre-dependent jRGECO1a. (**A**) eGFP expression driven by the *Pnoc* promotor (top), flp-dependent expression of ChR2-mCherry (middle) and co-localization (bottom) is shown at low (left) and high (right) magnifications corresponding to the boxed regions. (**B**) DAPI staining (top), cre-dependent expression of jRGECO1a in AgRP neurons (middle) and co-localization (bottom) is shown at low (left) and high (right) magnifications corresponding to the boxed regions. Scale bars are 100 μm. 3V, 3rd ventricle; a.c., anterior commissure.

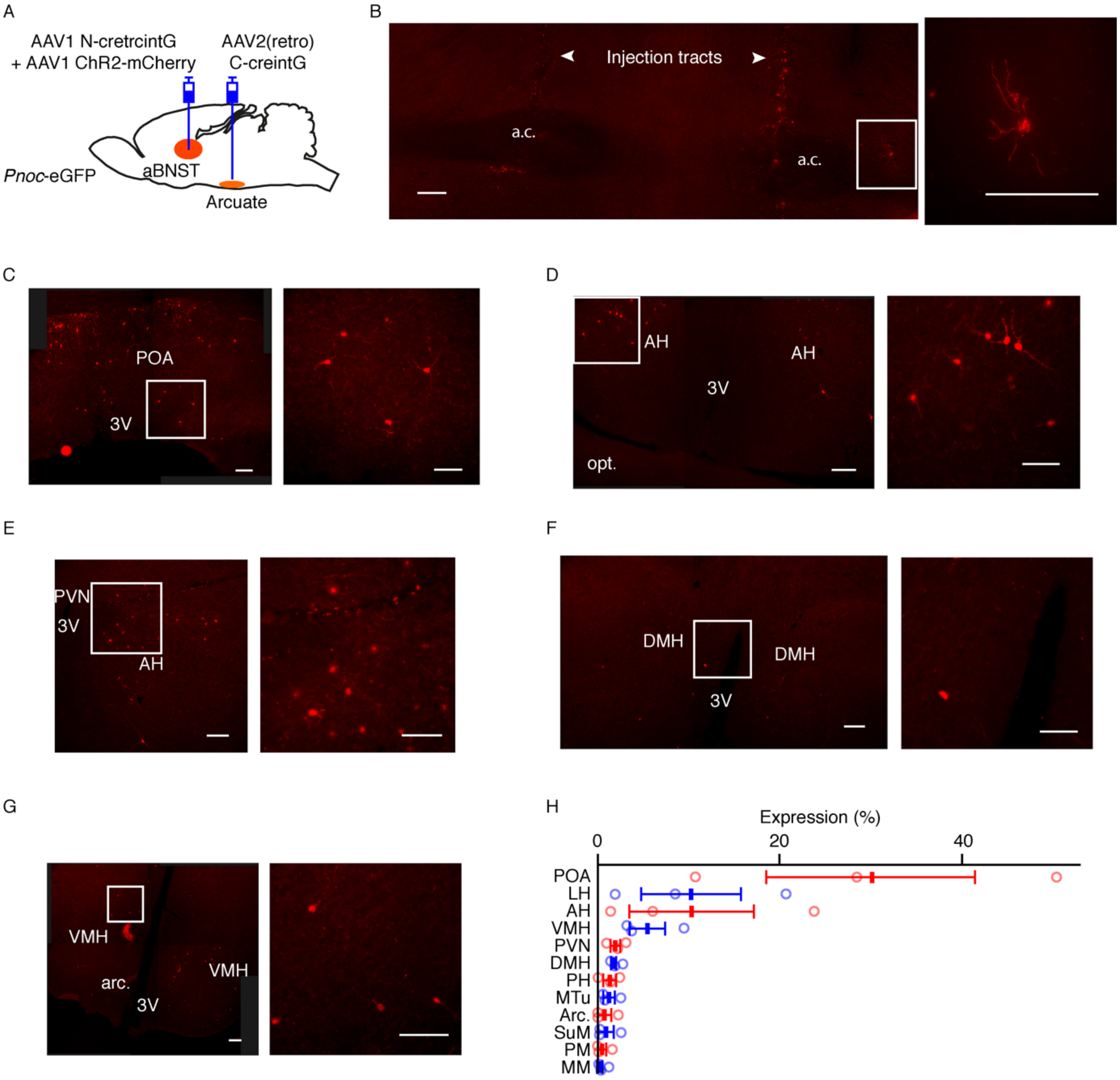

Supplemental Figure 8. A population of aBNST *Pnoc* neurons receives inputs from the hypothalamus.

(**A**) Diagram showing one component of DOGCre (C-creintG, serotyped with AAV2(retro) to enable retrograde expression) injected into arcuate nucleus and complementary component of DOGCre (N-cretrcintG serotyped with AAV1) injected into aBNST of *Pnoc*-eGFP mice to target aBNST *Pnoc* neurons projecting to mediobasal hypothalamus. (**B**) Cre-dependent ChR2-mCherry was injected into aBNST as an expression marker. Mosaic low magnification image (left) and expanded section (right) corresponding to the boxed region (n = 3). (**C**- **G**) Expression of mCherry driven by rabies virus in pre-synaptic neurons downstream from hypothalamic projecting aBNST *Pnoc* neurons in the POA (**C**), AH (**D**), PVN (**E**), DMH (**F**), VMH (**G**). Expanded sections (right) correspond to the boxed region in the mosaic low magnification images (left, n = 3). Scale bars are 200 μm and 100 μm for low and high magnification images, respectively. (**H**) mCherry expression levels in the hypothalamus as a percentage of the total number of cells expressing mCherry (excluding the BNST). Mean ± SEM from 3 mice. 3V, 3rd ventricle; a.c., anterior commissure; AH, anterior hypothalamus; Arc., arcuate nucleus; DMH, dorsomedial hypothalamus; LH, lateral hypothalamus; MM, medial mammillary nucleus; MTu, medial tuberal nucleus; opt., optic tract.; PVN, paraventricular nucleus; PH, posterior hypothalamus; PM, pre-mammillary nucleus; POA, preoptic area; SuM, supra-mammillary nucleus; VMH, ventromedial hypothalamus.

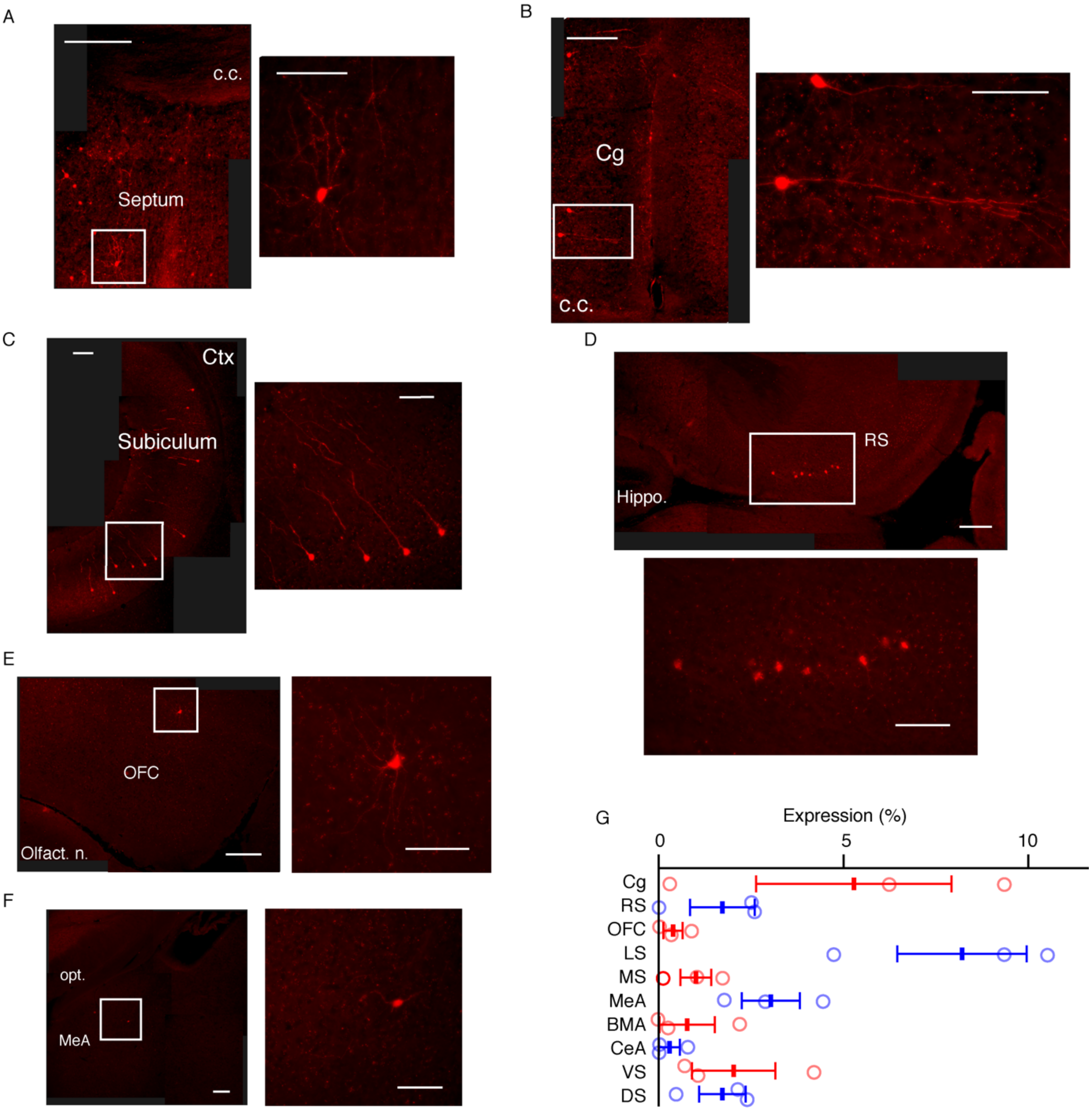

Supplemental Figure 9. Hypothalamic projecting aBNST *Pnoc* neurons receive presynaptic inputs from limbic and cortical regions.

(**A**- **F**) Expression of mCherry driven by rabies virus in pre-synaptic neurons downstream from hypothalamic projecting aBNST *Pnoc* neurons in the septum (**A**), cingulate (**B**), subiculum (**C**), retrosplenial cortex (**D**), orbital frontal cortex (**E**), medial amygdala (**F**). Expanded sections (right) correspond to the boxed region in the mosaic low magnification images (left, n = 3). Scale bars are 200 μm and 100 μm for low and high magnification images, respectively. (**G**) Expression of mCherry labelled neurons outside of the BNST and the hypothalamus. Mean ± SEM from 3 mice. BMA, basomedial amygdala; CeA, central amygdala; c.c., corpus callosum; DS, dorsal subiculum; Cg, cingulate cortex; hippo., hippocampus; LS, lateral septum; MeA, medial amygdala; MS, medial septum; Olfact. n., olfactory nucleus; opt., optic tract; OFC, orbital frontal cortex; RS, retrosplenial cortex; VS, ventral subiculum.
